# The Impact of Electric Fields on Processes at Electrode Interfaces

**DOI:** 10.1021/acs.chemrev.4c00487

**Published:** 2025-01-16

**Authors:** Zhuoran Long, Jinhui Meng, Lydia R. Weddle, Pablo E. Videla, Jan Paul Menzel, Delmar G. A. Cabral, Jinchan Liu, Tianyin Qiu, Joseph M. Palasz, Dhritiman Bhattacharyya, Clifford P. Kubiak, Victor S. Batista, Tianquan Lian

**Affiliations:** † Department of Chemistry and Energy Sciences Institute, Yale University, New Haven, Connecticut 06520, United States; ‡ Department of Chemistry, Emory University, Atlanta, Georgia 30322, United States; § Department of Chemistry and Biochemistry, University of California, San Diego, 9500 Gilman Drive, MC 0358, La Jolla, California 92093, United States; ∥ Department of Molecular Biophysics and Biochemistry, Yale University, New Haven, Connecticut 06520, United States

## Abstract

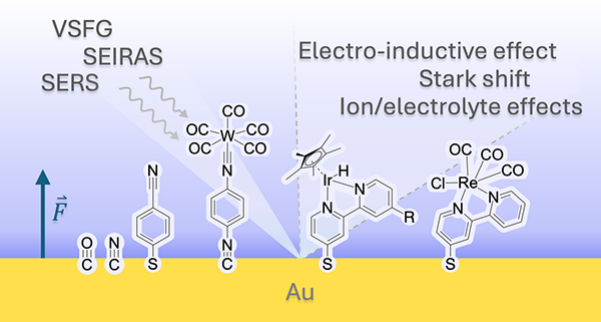

The application of external electric fields to influence chemical reactions at electrode interfaces has attracted considerable interest in recent years. However, the design of electric fields to achieve highly efficient and selective catalytic systems, akin to the optimized fields found at enzyme active sites, remains a significant challenge. Consequently, there has been substantial effort in probing and understanding the interfacial electric fields at electrode/electrolyte interfaces and their effect on adsorbates. In this review, we examine recent advances in experimental, computational, and theoretical studies of the interfacial electric field, the origin of the vibrational Stark effect of adsorbates on electrode surfaces, and the effects of electric fields on reactions at electrode/electrolyte interfaces. We also discuss recent advances in control of charge transfer and chemical reactions using magnetic fields. Finally, we outline perspectives on key areas for future studies.

## 1 Introduction: Electric Fields in Biomolecular Systems and at Electrode Interfaces

**Figure 1:**
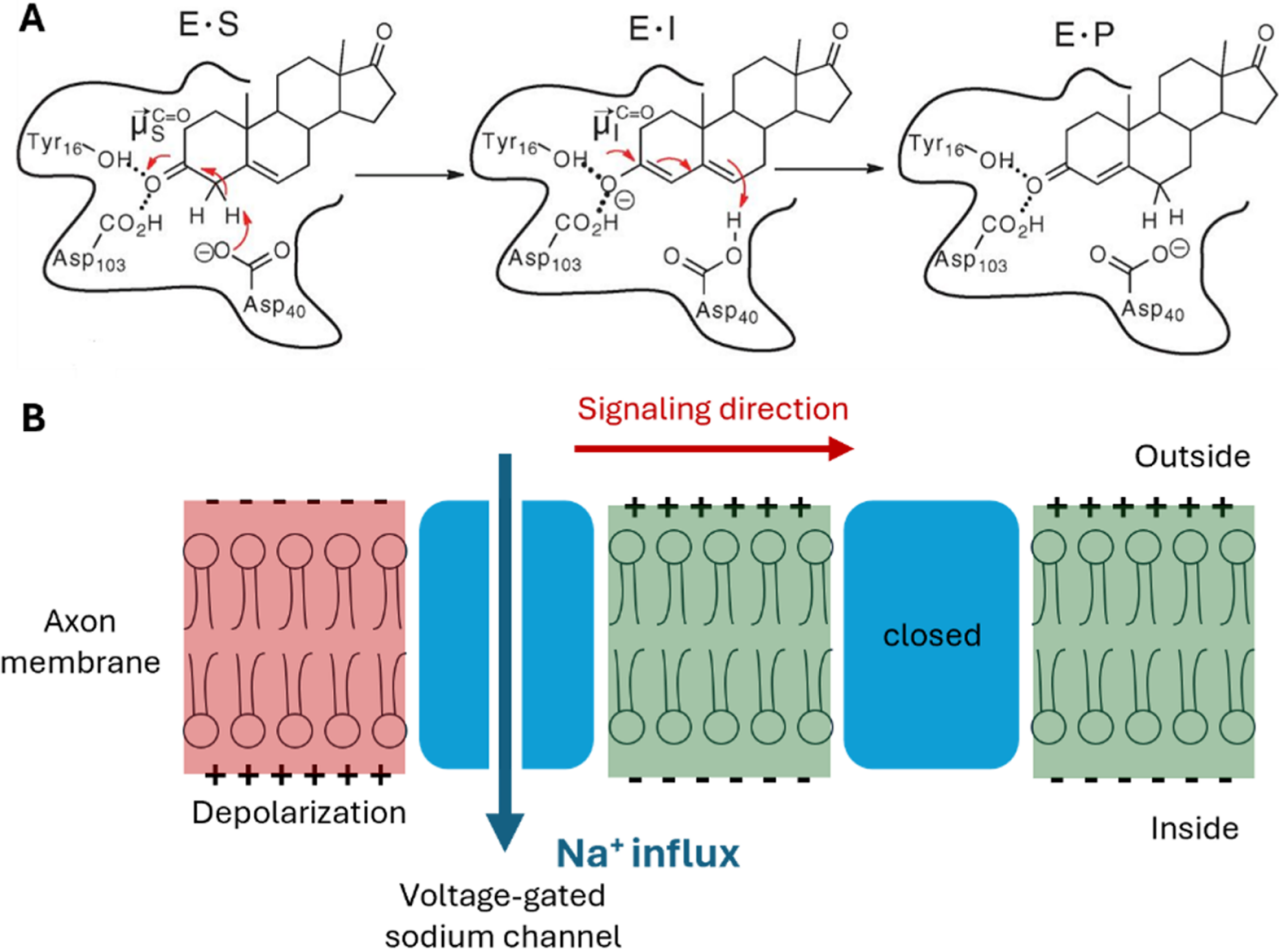
(A) Conjugated isomerization of the steroid 5-androstene-3,17-dione to 4-androstene-3,17-dione catalyzed by ketosteroid isomerase (KSI). The first step is the enolization and the second step is the reketonization of the carbonyl. (B) The voltage-gated sodium channels participating in the electric signal propagation along an axon. The initial depolarization opens the channel and results in an influx of sodium ions. This Na^+^ influx changes the local potential and induces the opening of the next channel, thus propagating the electric signal. (A) Adapted with permission from ref (3). Copyright © 2014, The American Association for the Advancement of Science.

Electric fields (EFs) formed by charged functional groups, ions in electrolyte solutions, or applied potentials are ubiquitous in the catalysis of natural and artificial systems. In enzymes, catalytically active sites often contain EFs generated by polar amino acid residues which lower the energy barriers of rate-limiting steps by stabilizing polarized transition states.^1−5^ For instance, the conjugated isomerization of steroids that occurs in aqueous solution is catalyzed by the enzyme ketosteroid isomerase (KSI) as shown in Figure 1A.^6^ The reaction rate increases from 6 × 10^–4^ M^–1^ s^–1^ in solution to 10^4^ ∼ 10^5^ M^–1^ s^–1^ when catalyzed by KSI due to two critical factors: (1) the precise positioning and orientation of aspartic acid (D40) that facilitates proton abstraction,^7^ and (2) the strong EF generated by Y61 and D106 that stabilizes the dienolate intermediate.^4,8^

Another example of the importance of EFs in biological systems is the potential created by a gradient of charged ions across a biological membrane. Proteins, such as voltage-gated sodium channels, respond to this EF to carry out essential functions, e.g., propagating electrical signals along axons (Figure 1B).^9,10^ When chemical signals are received, the membrane potential shifts, activating the voltage-sensing domain made up of four transmembrane alpha-helices with many charged residues. Through a “sliding-helix” mechanism,^11^ these helices move, changing the conformation of the pore gate domain and opening the channel to allow sodium ions to flow in. This influx triggers the opening of subsequent sodium channels, propagating the electrical signal along the axon.

**Figure 2:**
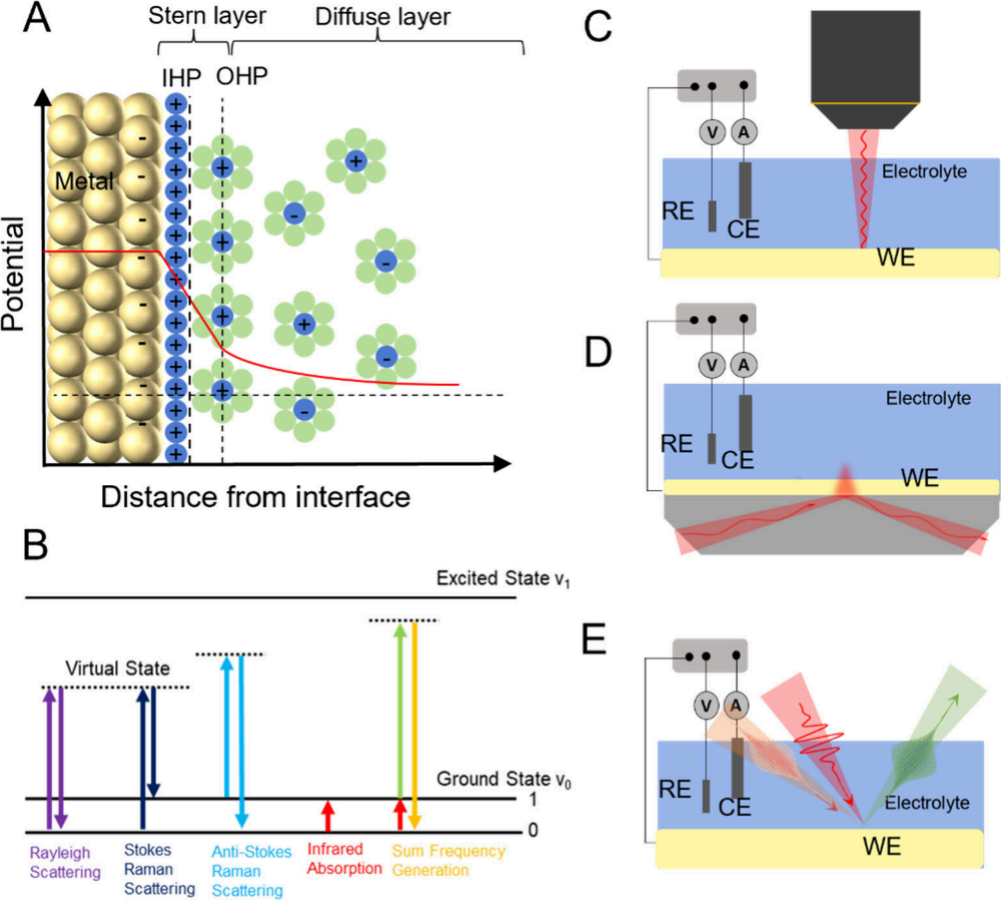
(A) Schematic of the electrical double layer (EDL) at a metal/electrolyte interface based on the Gouy–Chapman–Stern model. The inner Helmholtz plane (IHP) and the outer Helmholtz plane (OHP) are divided by the left dashed line, and the right dashed line divides the Stern layer and the diffuse layer. (B) The schematics of different light–matter interaction processes. The dotted lines stand for the virtual states, and the solid lines represent real states. Rayleigh scattering (purple) involves no energy change; Stokes Raman (dark blue) and Anti-Stokes Raman (light blue) represent inelastic processes where the energy shifts (Raman shifts) correspond to the vibrational modes of the molecules; infrared absorption (red) is directly related to certain vibrational modes of molecules; sum frequency generation (SFG, yellow) shows the nonlinear interaction of two photons resulting in a third photon with a summed frequency of the other two. (C) Schematic of the setup for electrochemical SERS. SERS-based techniques can probe molecular interfacial vibrational information under potential control by the surface-enhanced Raman scattering phenomenon, usually requiring the electrode surface to be modified by nanostructures active for surface plasmon resonance generation under laser excitation, e.g., Au/Ag/Cu nanoparticles for surface enhanced Raman spectroscopy (SERS) or Au core–shell nanoparticles for shell-isolated nanoparticle enhanced Raman spectroscopy (SHINERS). (D) Schematic of the electrochemical attenuated total reflectance (ATR)-SEIRAS setup. SEIRAS also probes molecular information at electrochemical interfaces similarly through surface enhancement by surface plasmon resonance on nanostructured metal thin film electrodes. (E) Schematic of spectroelectrochemical vibrational sum-frequency generation spectroscopy (VSFGS). VSFGS utilizes two temporally and spatially overlapped laser pulses to generate the third pulse for detection and intrinsically probes the interfacial molecular information with no nanoparticles needed.

In *artificial* systems, EFs are commonly induced at electrode–electrolyte interfaces in electrochemical systems. In such systems, when potential is applied to the working electrode, processes at the electrode interface can be divided into two categories: faradaic and nonfaradaic. Faradaic processes involve the transfer of electrons across the interface in the oxidation or reduction of chemical species. The amount of redox reactions is proportional to the number of electrons passed, i.e., faradaic processes follow Faraday’s law. Nonfaradaic processes involve current flow, but no electron transfer occurs across the interface. This current flow causes capacitive charging of the electrical double layer (EDL).^5,12−14^ A schematic of the Gouy–Chapman–Stern model of the EDL is illustrated in Figure 2A and further discussed in section 2.3. The charges on the electrode and the charges and dipoles of species in the EDL together generate the EF at the interface. While generated by nonfaradaic processes, such EFs influence both chemical and faradaic processes at electrode interfaces.

Unlike the sophisticated integration of EFs in biological systems, the deliberate application of EFs to manipulate microscopic processes in *artificial* systems is an emerging area of research. It is nontrivial to design and utilize these interfacial EFs for specific catalytic transformations at electrode interfaces. Immobilization of molecular catalysts on metal surfaces represents an outstanding challenge. In contrast to catalytic cofactors embedded in the active sites of enzymes where local electric fields lower the energetic barrier of rate limiting steps, homogeneous catalysts that work well in solution often exhibit unfavorable interactions when immobilized on electrodes, resulting in poor performance. Additional challenges include low catalyst loading (monolayer and submonolayer), synthesis of catalysts with surface-attachment moieties, control of adsorbate orientation, etc.^15^ These challenges may beaddressed by a more detailed understanding of immobilization effects, which may be achieved through *in situ* spectroelectrochemical characterization techniques to probe local EFs and the properties of adsorbates in EDLs, in collaboration with theoretical and computational studies of adsorbates at interfaces.

Herein, we review recent advances in the development and implementation of combined experimental and computational techniques to utilize EFs to affect the properties and reactivity of molecular adsorbates at interfaces and understand the underlying mechanisms. Vibrational spectroscopic tools for probing EDL structures, local EFs, and interfacial environments are reviewed in section 2. Commonly utilized and state-of-the-art computational methods to introduce EFs to model systems of electrode interfaces are summarized in section 3. Recent advances in the use of EF for characterization of reactive molecular adsorbates and interfacial mechanisms are reviewed in section 4. A discussion of the effect of magnetic fields (MFs) on chemical processes is presented in section 5. Finally, a summary and our perspective on future utilization of EF effects on chemical properties and reactions at electrode interfaces are provided in section 6.

## 2 Vibrational Spectroscopic Tools for Probing Electric Fields at Interfaces

### 2.1 The Stark Effect: Measuring Electric Fields at Interfaces by Vibrational Spectroscopy

The Stark effect refers to the change in the energy level splitting caused by the EF. This effect arises primarily from the difference in the dipole moments of a probe at various energy levels. The EF stabilizes energy levels with dipole moments parallel to the field and destabilizes energy levels with antiparallel dipole moments, thus altering the energy level splitting. By measuring this change, the magnitude and direction of the electric field can be inferred.^16^ Furthermore, analyzing the Stark effect on a given probe can provide valuable insights into the molecular polarizability, the dielectric environment, and the charge distribution around the probe. Here, we focus on the vibrational Stark effect^17^ that has been widely used to characterize local EFs which affect the properties and functionality of molecular systems at electrode/electrolyte interfaces, as well as to describe the profile of EFs at the EDL with subnanometer resolution.

In a vibrational Stark spectroscopic measurement^17−19^ experimental data is usually interpreted in the context of the conventional Stark equation:^18,20−23^
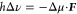
1where *h* is Planck’s constant, *v* the vibrational frequency of the vibrational probe, *h*Δν (*or ℏ*Δω) is the change of the vibrational energy, ***F*** the EF, and **Δμ** the Stark tuning rate which reflects the change in dipole moment associated with the vibrational mode. In most cases, the applied EF is considered to be a parallel vector field perpendicular to the electrode surface. If we use ***F*** to denote the EF strength at the region of the probe and Δμ for the projection of **Δμ** on the direction of EF, eq 1 can be rewritten into a scalar product format:
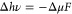
2From eq 2, we can derive the expression of the EF strength *F*(φ) as a function of potential φ (applied voltage):^22^
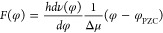
3where  represents the change in vibrational frequency with respect to the applied potential, *φ*_PZC_ is the potential of zero charge (PZC) representing the potential at which the net charge on the electrode surface is zero.  is measured by the experiment, and Δμ is obtained from computational methods. The experimentally determined Stark tuning slope of the vibrational mode can be used to determine the strength of the field felt by the mode at its position relative to the electrode and, thus, how rapidly the potential drops at that position. A common and confusing term pair for many researchers is the Stark tuning rate (Δμ) and the potential-dependent frequency shift .^24^ The former is an intrinsic property of the probe and is usually determined by the electronic structure of the conjugated molecular system which quantifies how the dipole moment of a certain molecule changes under an EF. The latter is often referred to as the Stark tuning slope, Stark shift slope, or frequency shift gradient, and is an experimentally measured parameter reflecting how the mode’s frequency responds to a change in the applied electrochemical potential, which can be linear or nonlinear. This term depends on the local interfacial environment and subjects to factors such as molecular orientation, surface coverage, etc., and therefore, it is usually not a fixed number and can vary dramatically under different experimental conditions.^25^

The vibrational Stark slope can be measured by various electrochemical *in situ* surface selective or surface enhanced vibrational spectroscopic tools to probe local EFs at the interfaces.^21−23^Figure 2B summarizes and compares the basic generation mechanisms and energy level differences of different light-matter processes, including Raman scattering, infrared absorption, and sum frequency generation. The spectroscopic tools derived from these processes either selectively enhance the signal from interfaces, as in surface-enhanced Raman spectroscopy (SERS, Figure 2C)^26,27^ and surface-enhanced infrared absorption spectroscopy (SEIRAS, Figure 2D),^21,28,29^ or are intrinsically generated from the surface^7^ as in vibrational sum frequency generation spectroscopy (VSFGS, Figure 2E).^22,24,30^ These vibrational spectroscopic techniques are applied *in situ* as illustrated in Figure 2C–E, all of which are typically integrated with a standard three-electrode electrochemical setup. In a SERS setup (Figure 2C), a laser is used to generate Raman scattering at the working electrode, which usually leverages plasmonic enhancement from nanostructured metallic surfaces or drop-cast nanoparticles to enhance the Raman signals of the molecules near the surface. In the SEIRAS setup (Figure 2D), the working electrode is prepared on an attenuated total reflection (ATR) prism, and infrared light is directed at the surface. The enhancement of the absorption is similar to SERS, where the metallic surface’s roughened morphology also leads to plasmonic resonance, and an enhanced IR signal is obtained. In the third case, VSFGS (Figure 2E), two pulsed laser beams interact and overlap spatially and temporally at the working electrode surface, with one beam typically in the infrared range and the other in the visible region; the nonlinear process at the electrode surface generates a third pulse, i.e., the sum frequency signal, which is detected and used for vibrational information analysis. Each of the techniques (SERS, SEIRAS, and VSFGS) can be used to probe interfacial molecular information, but they also differ in specific applications. In surface-enhancement-based techniques, such as SERS and SEIRAS, their signals are dominated by the subpopulation of the surface/interface molecules at the “hot spot” where the electromagnetic fields are enhanced; they require plasmonic nanostructures or nanoparticles (NPs) near the surface to enhance the intensity of the interfacial optical phenomena.^26,27,31^ Their signals also contain unenhanced contributions from molecules in the bulk, beyond the EDL, due to their large number, which can often be subtracted to reveal only the surface/interface contributions. Thus, both techniques are critically dependent on the substrate preparation in terms of metal (Au, Ag, etc.) and nanostructure sizes. VFSGS, on the other hand, does not have strict requirements for the substrate nanostructure morphology. Though the signal can also be enhanced at plasmonic substrates, it can intrinsically probe the behavior of the majority of molecules at interfaces without the enhancement-based substrates because it is a nonlinear optical technique.^32^ VSFGS can also provide additional molecular information, such as orientation and structural information, through analysis of the combination of different polarizations of incident and collected beams, information which is difficult to obtain by SERS or SEIRAS.^33,34^

In the following sections, we review vibrational spectroscopic studies of the EDL at electrochemical interfaces as compared to theoretical models of the EDL, including the Gouy–Chapman model, Gouy–Chapman–Stern model, and EDL descriptions obtained by molecular dynamics simulations.^22,35^ We also discuss recent progress in the field focused on various molecular systems, with an emphasis on understanding EDL environments surrounding molecular adsorbates at electrode/electrolyte interfaces.

### 2.2 Probes of Electric Fields: CO, CN^–^, and SCN^–^ on Metals

The development of SERS by Fleischmann et al.^36^ and Jeanmaire et al.^37^ followed by SEIRAS enabled the detection of vibrational signals of adsorbed molecules and ions at the electrochemical interfaces. Since then, experimentalists have utilized adsorbates with strong vibrational bands, such as CO, CN^–^, or SCN^–^ to probe EF in the double layer.

**Figure 3:**
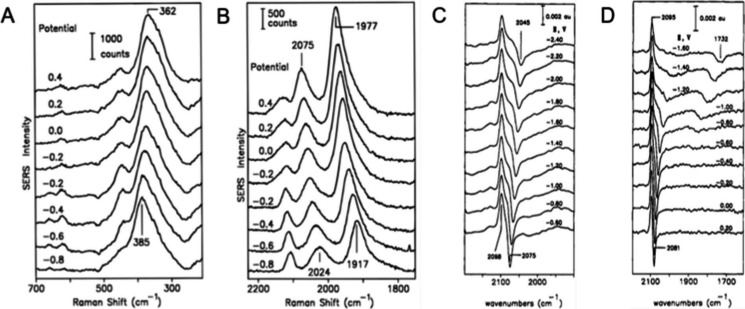
Potential dependent SERS (vs SCE) of CO on Pd electrode in (A) low frequency and (B) high frequency region. Spectra from −0.8 to −0.2 V were obtained in CO-saturated 0.1 M KClO_4_ + 0.01 M KOH; spectra from −0.2 to 0.4 V, were obtained with 0.02 M HClO_4_ added. Potential-difference infrared (PDIR) absorbance spectra of CO on Pt in CO-saturated acetonitrile containing (C) 0.15 M THAP and (D) 0.15 M NaClO_4_ (vs Fc^+/0^). (A,B) Adapted with permission from ref (40). Copyright 1992 American Chemical Society. (C,D) Adapted with permission from ref (41). Copyright 1996 American Chemical Society.

Pioneering studies in this area come from Weaver and co-workers, who conducted a series of studies of CO and other adsorbates on different metal surfaces in acidic and neutral electrolyte media by SERS (Figure 3A,B) and SEIRAS (Figure 3C,D).^38−43^ They reported the vibrational frequencies of CO on metals ranging from ∼1700 to ∼2100 cm^–1^, depending on their relevant binding affinity on the metal, as well as their potential-dependent frequency shift slopes, ranging from ∼10–20 cm^–1^/V to ∼30–60 cm^–1^/V.^38,40,41^

**Figure 4:**
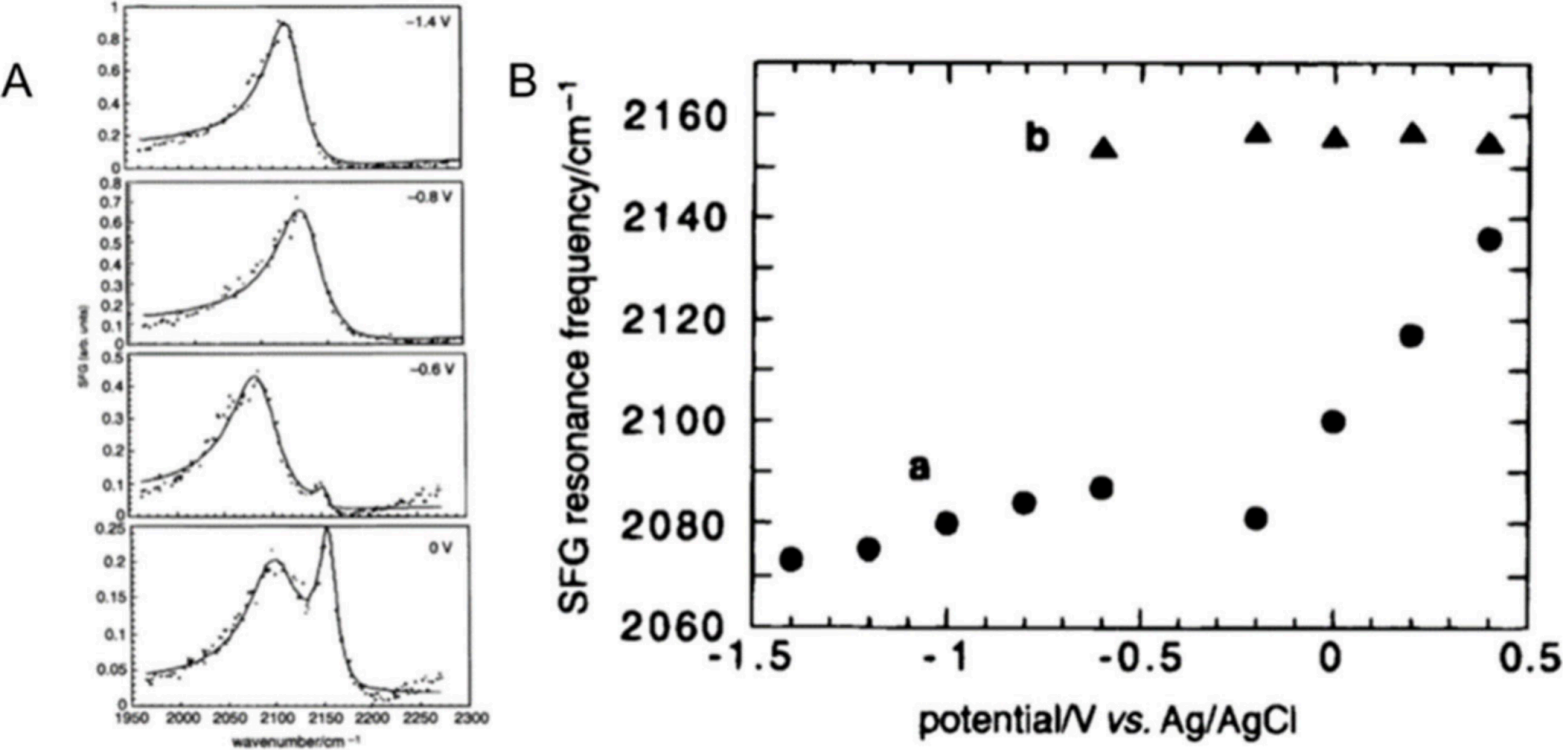
(A) Potential dependent VSFG spectra of Pt(110) in contact with a solution containing 0.1 M NaClO_4_ and 0.025 M KCN. Dots represent experimental values, and continuous lines are the fit of the experimental spectra. (B) Variation of VSFGS resonance frequency as a function of the electrode potential: (a) and (b) represent the frequencies corresponding to the broad (N-end adsorption) and sharp (C-end adsorption) transitions, respectively. The Stark tuning slope of the low frequency peak changes drastically with the appearance of the high frequency (C-end adsorption) peak. Adapted with permission from ref (56). Copyright 1996 Royal Society of Chemistry.

Guyot-Sionnest and Tadjeddine first applied SFG spectroscopy to study the adsorption of CN^–^ and SCN^–^ anions at the polycrystalline platinum electrode.^34,44−54^ There were two absorption bands observed that correspond to the N-bound and C-bound species of CN^–^ at the platinum surface (N-bound and S-bound for SCN^–^).^55^ In later experiments,^45,56^ similar CN^–^ absorption features were found on the surface of Pt single-crystal electrode, suggesting that the adsorption properties on the electrode surface are genuine, and not induced by the surface defects of the polycrystalline electrodes. The absorption peak positions, the width of the resonances, and the overall shape of the spectra, however, are affected by the nature of the polycrystalline surface, along with the presence of various surface defects. Tadjeddine et al. investigated the binding features of CN^–^ on a Pt(110) single-crystal electrode.^56^ As shown in Figure 4, in the potential region of hydrogen evolution (−1.4 V vs Ag/AgCl), one broad asymmetrical resonance is observed with a peak at ∼2070 cm^–1^ and a width of ca. 45 cm^–1^, corresponding to the adsorption of CN^–^ through the nitrogen end. Changing the potential from −1.4 V to −0.6 V induces a Stark shift to this adsorption peak by ∼20 cm ^–1^/V with a slight increase in the full width at half-maximum (FWHM) and negligible change in peak amplitude. A second peak appears at ∼2150 cm^–1^ when the electrode potential becomes more positive. This resonance is considerably narrower and corresponds to the C-end adsorption on the Pt surface. As the electrode potential is driven to more positive values, the peak continues to grow in intensity, and the peak frequency appears to be independent of the electrode potential. However, the Stark tuning slope of the low-frequency peak (∼2070 cm^–1^, N-end adsorption) is drastically changed from 20 cm ^–1^/V to 90 cm ^–1^/V as soon as the C-end adsorption (∼2150 cm^–1^) begins to take place. The potential-dependent SFG spectra of the adsorption of CN^–^ on polycrystalline Ag electrode (in contact with 0.1 M NaClO_4_ and 0.01 M KCN) has also been studied by Tadjeddine et al.^54^ The absorption band is centered at ∼2110 cm^–1^ and experiences a Stark shift of 20 cm^–1^/V, in accordance with the previously measured IR and Raman spectra.^57,58^

These observations of the frequency shift of the adsorbates at interfaces under applied electrostatic field were further interpreted and elucidated via modeling and experimental studies.^42,59,60^ One early explanation suggested that the varied adsorbate coverage under different potentials was the origin of the changing frequency, which would lead to a different dipole–dipole coupling among molecules through dipole fields.^61,62^ However, Lambert et al. later showed that the origin of observed vibrational frequency shifts matches well with theoretical predictions both by the vibrational Stark effect, i.e., from the adsorbates’ interaction with the EF through space, and by the effect of the potential on the occupancy of the adsorbates’ antibonding orbitals.^59,60^ Further theoretical study by Head-Gordon and Tully demonstrated the importance of charge transfer interaction between CO and Cu on their field-dependent CO stretching frequency.^63^ Subsequent work by Weaver and co-workers illustrated that both of the mechanisms proposed by Lambert contribute to the frequency shift.^42^ Therefore, the term vibrational Stark effect (VSE) was proposed to include both contributions from the EF and the effect of the potential on the adsorbates’ antibonding orbitals.

A more recent study where CO molecules are densely packed close to the interface of Pt showed that the oxidation onset potential and Stark tuning slope of the CO stretching mode was independent of electrolyte concentration (and therefore diffuse layer thickness) in an aqueous solution with varying amounts of perchloric acid.^64^ This description of the potential drop represents a “special double layer structure” distinct from the conventional Guoy–Chapman model discussed in detail in the next section.^64^ The authors’ proposed explanation is that there is modest potential drop across the CO stretch and that the majority of the potential drop occurs between the O end of the molecule and the OHP due to hydrophobic interactions with the supporting electrolyte. In recent years, however, studies have shown that the vibrational Stark shift of weakly bonded adsorbates (such as weakly bonded CO or anions) on electrodes may originate from effects other than EF changes, such as morphology changes of surfaces,^65^ coverage changes of adsorbates,^66^ or even probe technique effects.^43,67^ Thus, more strongly bonded interfacial systems with self-assembled monolayers (SAMs) are more reliable and widely used in studying the interfacial EFs. In the next section, vibrational Stark spectroscopy studies with a variety of SAMs are discussed in relation to the characterization of the double layer.

### 2.3 Utilization of Experimental Vibrational Stark Probes to Validate Theories on Electrical Double Layers

The electrical double layer (EDL) refers to the manner in which ionic charge is distributed near the interface of a charged surface in contact with a liquid electrolyte.^68^ Over time, the picture of the EDL was revealed through theoretical modeling, followed by verification in a series of experiments. A comprehensive introduction and review of different models of the EDLs at electrochemical interfaces can be found in Bard and Faulkner’s book.^35^ The EDL refers to the interface between a charged surface and an electrolyte solution. It consists of two distinct regions: the inner Helmholtz plane (IHP) and the outer Helmholtz plane (OHP) (Figure 2A). The IHP is a narrow region in immediate proximity to the charged surface, where ions are strongly attracted due to electrostatic interactions. The OHP, on the other hand, extends further into the solution and is characterized by a lower concentration of ions. Various models have been proposed to describe the electrical double layer and its properties. One such model is the Gouy–Chapman model, which assumes that the ions are distributed in a diffuse layer near the charged surface. This model considers the electrostatic repulsion between ions and the role of thermal energy in determining the distribution of ions in the double layer. Another model, known as the Stern model (or the Gouy–Chapman–Stern model), considers the specific adsorption of ions at the charged surface, leading to the formation of an inner compact layer along with a diffuse layer.

Traditionally, electrochemists relied on techniques like cyclic voltammetry and electrochemical impedance spectroscopy (EIS)^71^ to provide experimental support for different models of the EDL. However, with the establishment of the correlation between interfacial EF and the vibrational Stark effect by pioneering work described in the previous section, vibrational spectroscopic techniques have also been employed now to study the structure of the EDL and validate different theoretical models.

**Figure 5:**
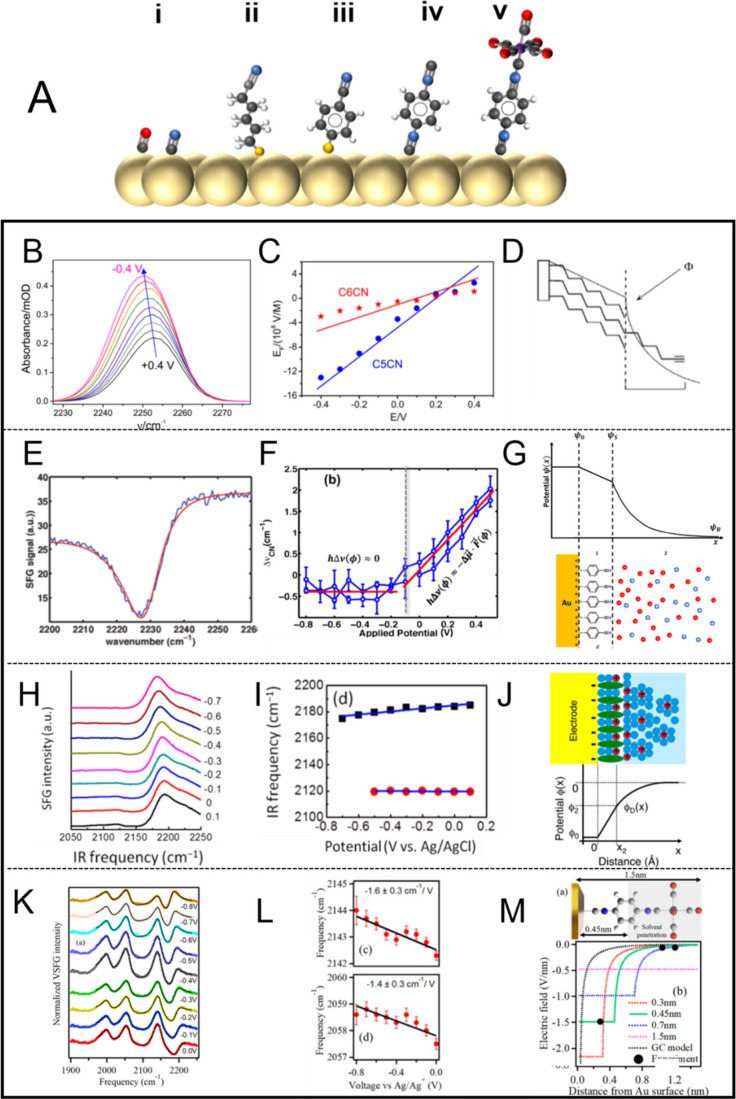
(A) Different SAMs/molecules used for EF studies at electrochemical interfaces: (i) CO or CN^–^; (ii) alkylthio nitrile terminated SAMs; (iii) 4-MBN SAMs; (iv) diisocyanide SAMs; (v) tungsten-pentacarbonyl(1,4-phenelenediisocyanide) complex. Atom colors are as follows: light yellow, Au (or Pt), black-C, red-O, blue-N, white-H, yellow-S, purple-W. (B–M) Different lines show the development of the studies on the EDL structure with SAM on metal. Row 1 with B, E, H, and K shows the experimental spectroscopic data; row 2 with C, F, I, and L shows the Stark tuning slopes of certain modes; row 3 with D, G, J, and M shows the schematics of the model and the potential or EF drops. (B,C) ATR-IR study based on alkylthiol SAMs terminated with nitrile groups, the corresponding spectra, and proposed EF profile in different length SAM systems. (D) Potential drop graphical depiction of the interfacial boundary region similar to the study in B and C. (E–G) SFG study based on 4-MBN SAMs, represented spectra and the proposed electric potential profile in the system. (H–J) SFG study based on diisocyanide SAMs, the corresponding spectra and proposed electric potential profile of the SAM system. (K–M) SFG study based on the molecular ruler system, the corresponding spectra and proposed electric potential profile. (B,C) adapted with permission from ref (28). Copyright 2017 American Chemical Society. (D) Adapted with permission from ref (69). Copyright 2003 American Chemical Society. (E–G) Adapted with permission from ref (70). Copyright 2017 American Chemical Society. (H–J) Adapted with permission from ref (30). Copyright 2017 American Chemical Society. (K–M) Adapted with permission from ref (22). Copyright 2022 American Chemical Society.

To probe the EDL, self-assembled monolayers (SAMs) of organic molecules on metal surfaces provide well-defined, stable, and spectroscopically accessible systems with relatively consistent coverage during the potential change. As shown in Figure 5A, many notable examples from these spectroscopic studies are based on the strong vibrational modes of SAMs containing nitrile (−CN), isocyanide (−NC) and carboxyl (−CO) groups. These modes in different molecules usually provide a pronounced peak in both IR and Raman and are sensitive to the local EF changes in the EDL region and thus can be widely applied to different SERS/SEIRAS/VSFGS studies to validate the EDL theories. For instance, to provide direct experimental observation to verify the Gouy–Chapman–Stern model, a series of nitrile-terminated SAMs with different lengths were prepared on Au and Ag^28,69,72^ and used to track how the vibrational mode at different positions relative to the boundary between the Stern layer and diffuse layer responds to the change in ionic strength of the diffuse layer (Figure 5A(ii)).

**Figure 6:**
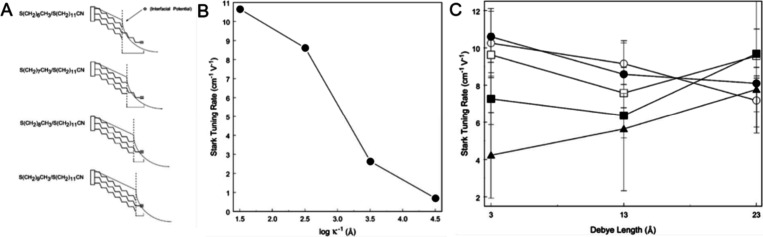
(A) Graphical depiction of the interfacial boundary region for each mixed-monolayer system within the diffuse layer. (B) Measured Stark tuning slopes for −S(CH_2_)_10_CH_3_/–S(CH_2_)_11_CN mixed monolayers as a function of Debye lengths (or decreasing ionic strengths). (C) Spatial profile of nitrile Stark tuning slopes as a function of the Debye length. Mixed SAMs with the same length of nitrile terminated SAM and different length of methyl terminals (HS(CH_2_)_*x*_-CH_3_, *x* = 6–10): solid triangle *x* = 6; solid square *x* = 7; hollow square *x* = 8; solid round *x* = 9; hollow round *x* = 10. Adapted with permission from ref (69). Copyright 2003 American Chemical Society.

In two of the studies, the authors utilized a mixed-SAM method to constrain the relative position of the nitrile probe moiety in the EDL.^69,72^ The densely packed alkanethiol SAM with methyl-terminals (HS(CH_2_)_*x*_-CH_3_, *x* = 6–10) serves as the position for the beginning of the diffuse layer and those with nitrile groups as the vibrational reporter for interfacial EF (Figure 6A). By changing the length of the methyl-terminated SAMs, the nitrile probe reflects the EFs at different spatial positions within the EDL, from close to the boundary to far from the boundary (far within the diffuse layer). Their major experimental results agree with the −Chapman theory, where the Stark tuning slopes and interfacial EFs depend on both the distance of the probe moiety and the ionic strength of the local environment. However, they also reported that the EDL extends further into the solution than predicted by the Gouy–Chapman theory, which the authors attributed to the effect of a hydrophobic interface of the adjacent water layer and the finite size of the solvated ions.

From their results, the authors provide a comprehensive picture of how the probe moiety responds to the ionic strength (Figure 6B,C). For the probe moiety positioned in the inner region of the diffuse layer (outside of the OHP, for mixed SAMs with *x* = 9, 10), as the ionic strength increases and the Debye length shortens, this confined space of the diffuse layer generates a larger potential gradient, i.e., the EF, leading to a positive tuning response of the probe moiety to the ionic strength (negative to the Debye length). For the probe moiety residing within the diffuse layer (SAMs with *x* = 6), at low ionic strength (larger Debye length), the EDL extends to tens of angstroms, producing a significant Stark tuning effect. When the ionic strength increases (shorter Debye lengths), the EDL compresses, and the EF at the probe moiety decreases, resulting in a smaller Stark tuning slope.

Similarly, the probe moiety within the diffuse layer can directly feel the ionic strength change and be used as evidence to support the theories on EDL.^23,70^ Another example study with spatial resolution is from Staffa et al. (Figure 5B–D).^28^ Using SEIRAS to probe different lengths of nitrile-terminated SAMs (C_5_CN and C_6_CN), the authors mapped the spatial profile of the EFs within the diffuse layer, which is applicable for analyzing the effects of EFs in biological processes at electrode/SAMs interfaces. Dawlaty and co-workers demonstrated another set of molecules with electrochemical VSFGS.^70^ The authors employed a SAM of 4-mercaptobenzonitrile (4-MBN) on Au to monitor the Stark shift of its nitrile stretch (Figure 5A(iii),E–G). Combined with capacitive studies, they find that the measured local field scales with the ionic strength, which again supports the Gouy–Chapman theory. Interestingly, they also observe that at positive potentials with negligible currents, the probe of the local field experiences a frequency shift. When the potential is swept in the negative region where electron transfer (ET) occurs (where the current is nonzero), the probe frequency does not change. The proposed explanation for this is that when passing charge, the interface no longer maintains a large potential drop, and therefore, the EF is no longer dependent on the applied potential. This is a direct illustration of the difference between how nonfaradaic and faradaic processes at the interface impact the local EF, and reveals that in an electrochemical reaction scheme with a sustained current flow, an increase in thermodynamic driving force does not necessarily translate to increased molecular polarization at the interface.

The concept of measuring vibrational modes at different positions of the SAMs to study the EDL was adopted in further systems. Ge et al. investigated the interfacial EDL structure and the EF at Au/diisocyanide/aqueous electrolyte interfaces utilizing a series of SAMs of three aromatic diisocyanides: 1,4-phenylene diisocyanide (PDI, Figure 5A(iv)), 4,4′-biphenyl diisocyanide (BPDI), and 4,4″-*p*-terphenyl diisocyanide (TPDI).^30^ As shown in Figure 5H–J, these SAMs were assembled on Au electrodes and monitored with potential dependent VSFGS and density functional theory (DFT) calculations. The results reveal that Au bound -NC and free -NC stretching modes show different potential-dependent slopes. Comparison of the stretching modes with DFT calculated Stark tuning rates allowed the quantification of the EF strength at each position. The detailed analysis of the Stark tuning slopes provides insight into the effective thickness of the Helmholtz layer, indicating negligible electrolyte penetration for PDI SAMs but significant penetration for BPDI and TPDI SAMs. DFT calculations show that the Stark tunning rate of the metal-bound NC is much larger than the free NC, suggesting important role of the metal binding on the polarizability of the NC bond, suggestive of the electro-inductive effect (discussed in more detail in section 2.5 and section 4). A more recent study by Bhattacharyya et al.^22^ introduced a novel vibrational Stark shift “ruler” molecule with multiple carbonyl (−CO) and isocyanide (−NC) groups (Figure 5A(v)) to map the EF strength across the EDL with subnanometer spatial resolution. The authors utilized a SAM with a tungsten-pentacarbonyl(1,4-phenelenediisocyanide) complex attached to a gold surface as the Stark shift ruler, where −CO and −NC moieties at different positions reported the relative changes in EF strength at set distances throughout the molecule by measuring Stark shifts. The results, as shown in Figure 5K–M, also support the Gouy–Chapman–Stern model, revealing a detailed Stern layer thickness of ∼4.5 Å, indicating substantial solvent and electrolyte penetration within the SAM, with a significant electro-inductive effect also observed on the W center located ∼1.2 nm away. To extend these studies beyond the limited potential allowed by conventional thiol or isocyanide based SAMs, Palasz et al. recently developed a new carbene-based SAM platform with more resilient stability to functionalize metal interface with −CO group, which enables probing the EDL with more extreme potentials, including with potential applications in catalysis.^73^

### 2.4 Applications of Vibrational Stark Probes to Study Molecular Details of Electrical Double Layers

There have been questions surrounding the origin of the interfacial EF effects on these surface-bound probes. Along with inductive effects (to be discussed in detail in section 2.5) on surface-bound probes, it is important to map inhomogeneity in the experienced field and understand the experimental conditions that give rise to it. Note that EDL models, such as the Gouy–Chapman–Stern model, although critical for advancing our understanding of electrode–solution interfaces, are mean-field theories that characterize the interfacial behavior in terms of macroscopic parameters (permittivity, salt concentration, and monolayer thickness) and therefore completely neglect the microscopic and dynamic nature of electrochemical interfaces. The latter can give rise to very complex phenomena involving solvent and ion organization of the double-layer, nanoscale structural and dynamic monolayer heterogeneity, and monolayer permeability. These phenomena, in turn, fine-tune the rate and selectivity of field-driven reactions at electrode–electrolyte surfaces. Several studies have probed this question by changing these parameters computationally and experimentally. Especially, atomistic computational studies of electrode–electrolyte interfaces can provide molecular-level details of these interfaces beyond the capabilities of mean-field theories.^74−83^

In a recent study, periodic DFT calculations were performed in combination with a multilayer solvation model to characterize the interfacial EF as a function of applied potential probed by the CN stretching of 4-MBN attached to a gold electrode.^84^ Their results displayed substantial spatial inhomogeneity of the interfacial EF and electrostatic potentials across the entire 4-MBN monolayer, showing oscillations in the region of the CN molecular probe. Similar conclusions were reached in the aforementioned study by Ge et al. in their vibrational Stark spectroscopy analysis of the diisocyanide SAMs series.^30^ In that work, atomistic molecular dynamics (MD) simulations were performed to characterize the electrode-SAM-electrolyte interfaces at the molecular level. Interestingly, the electrostatic profiles obtained from the MD simulations reveal oscillatory and inhomogeneous behavior, characterized by intercalating ions and water in the SAM interface.

While the local EF is a major contributor to the vibrational Stark shift of molecular reporters such as CO_ad_, 4-MBN, etc., the frequency and the line width of these molecules are also quite sensitive to the local environments, such as solvent effects, hydrogen bonding, and ion interactions. For instance, some studies discussed in previous sections show that the dielectric constant^85^ or the average solvent EF^16^ of certain solvents can be used to predict the vibrational frequency shift of the reporter molecule. It has also been shown that the local interfacial EF are modulated by different electrolytes.^14,86−89^ Baumberg and co-workers studied the effects of electrolyte penetration into the EDL with the shift of 4-MBN stretches.^23^ After their initial study of 4-MBN, they used a mixed-monolayer with 4-biphenylthiol to modulate the SAM, essentially protecting the nitrile group from ions in the bulk electrolyte. They reported a significant decrease in the shift in the nitrile stretch with increasing 4-biphenylthiol, illustrating that ion interaction with the SAM contributes significantly to the experienced field. Recently, Voegtle et al.^90^ and Sarkar et al.^91^ further studied how different ions from ionic liquids (ILs) and surfactants affect the interfacial EF with a vibrational Stark probe. It is also worth noting here that the application of vibrational Stark spectroscopy has been extended to study the EF effects in proteins/enzymes.^92,93^

Other studies took advantage of the cutting-edge spectroscopic tools, such as time-resolved vibrational spectroscopies or 2D vibrational spectroscopies to study the dynamics of solvent molecules or hydrogen bonds at the electrode/electrolyte interfaces.^94−97^ These studies also used CO_ad_ or 4-MBN as the local probe molecules, focusing on their interaction with water (solvent) molecules, where the hydrogen-bonded and non-hydrogen-bonded vibrational modes (i.e., nitriles) can be differentiated in the spectra and analyzed to obtain information on potential dependent dynamics.

The results presented here illustrate how monolayer character and electrolyte effects alter the EFs felt by vibrational probes in SAMs, and that the local environment of these surface-bound probes cannot completely be described by mean-field models, emphasizing that the interfacial environment surrounding surface-bound species is rich with areas for tunability.

### 2.5 Applications of Vibrational Stark Probes to Study Local Electric Fields: Basis for Reactivity

Besides the validation of EDL theories, vibrational Stark spectroscopies provide a direct and accurate method in reporting the local EF in different electrode–electrolyte interface environments for EF-controlled reactions.^16,72,85−88,90−92^ Specifically, measurement of the vibrational modes of precasted SAMs^98^ or molecular reactants/catalysts themselves^24,99^ at the interfaces can provide local EF information for hydrogen evolution reactions (HER),^98^ proton-coupled electron transfer (PCET) reactions,^99^ CO_2_ reduction reactions (CO_2_RR),^24,86^ etc. The effect of EF on the reaction catalyst can be quite staggering. For example, for the CO_2_RR catalyst thiol-substituted M(bpy)(CO)_3_X (M = Re, Mn, X = Cl, Br) immobilized on Au, the authors quantified the interfacial EF strength at the metal site to be on the order of 10^8^–10^9^ V/m for both Re and Mn catalysts, combining the Stark tuning slopes observed by VSFGS and DFT calculations.^24^

**Figure 7:**
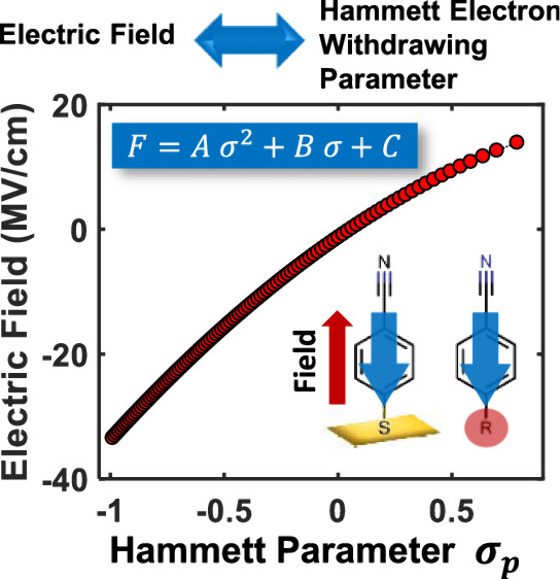
Relationship between applied EF and Hammett parameter established using 4-MBN. *A* = −3.94 ± 2.75 cm^–1^, *B* = 9.90 ± 1.82 cm^–1^, and *C* = 2349.5 ± 1 cm^–1^. Adapted with permission from ref (21). Copyright 2019 American Chemical Society.

Such strong vibrational frequency shifts prompt interest in understanding how interfacial EFs affect the reactivity of substrates or catalysts immobilized on electrode surfaces. In homogeneous systems, reactivity is often controlled using judicious ligand design based on the ligand’s electron withdrawing/donating capability, quantifiable by the Hammett parameter (σ_p_).^100^ Synthetic chemists utilize these substituents to modulate the electronic structure and properties of the molecules. A seminal study by Dawlaty and co-workers^21^ reported a correlation between an applied EF at an electrode interface and the Hammett parameter. Using VSFGS, the vibrational frequencies were monitored for the nitrile stretch of a monolayer of 4-MBN immobilized on a gold electrode. The Stark shift of the nitrile stretch versus applied bias was then compared to the FTIR shift of the nitrile stretch of a series of para-substituted benzonitrile molecules with R groups spanning −0.83 ≤ σ_p_ ≤ +1.11. From experimental results and computational analysis, the relationship between applied bias at an electrode and the Hammett parameter was determined to fit to a second-order polynomial (Figure 7).

There is some debate in the literature surrounding what gives rise to these effects and the terminology with which to present them. As discussed in section 2.2, a similar debate occurred previously on the origin of the vibrational Stark effect of CO on electrodes,^42,59,60,63^ through which it is generally accepted that charge transfer interaction between the electrode and CO plays an dominating role. More recently, the terms “electrodes as polarizing groups”^21^ and “electro-inductive effect”^15^ were introduced to refer to the change in the electronic structure of immobilized molecules as a result of an electric field created by applied bias on the electrode. These descriptions generally agree with what other researchers describe as the vibrational Stark effect,^101^ without explicitly separating the through-bond inductive effects (i.e., the electrode/adsorbate charge transfer interaction) and through-space field effect. More recent studies use the term electro-inductive effect to refer mostly to through-bond inductive effects.^102−104^ One such study addressed the question of whether the EF effects are “through-space” or “through-bond” by comparing the nitrile stretches of 4-MBN, which exhibits conjugation through the entire molecule, and 2-(4-mercaptophenyl)acetonitrile, which breaks conjugation before the nitrile stretch.^103^ Periodic DFT and *in situ* SERS were utilized to analyze the effect of breaking this conjugation. It was found that when the molecule remained conjugated, the nitrile stretch was affected strongly by through-bond induction, while the bent molecule’s shift was dominated by through-space EF effects. A follow up study systematically investigated breaking conjugation in a series of benzonitrile species, again showing that the greatest electro-inductive effect is observed for species with continuous conjugation to the electrode.^104^ These studies definitively illustrate that through-bond inductive effects play a significant role in the polarization of surface-attached species.

**Figure 8:**
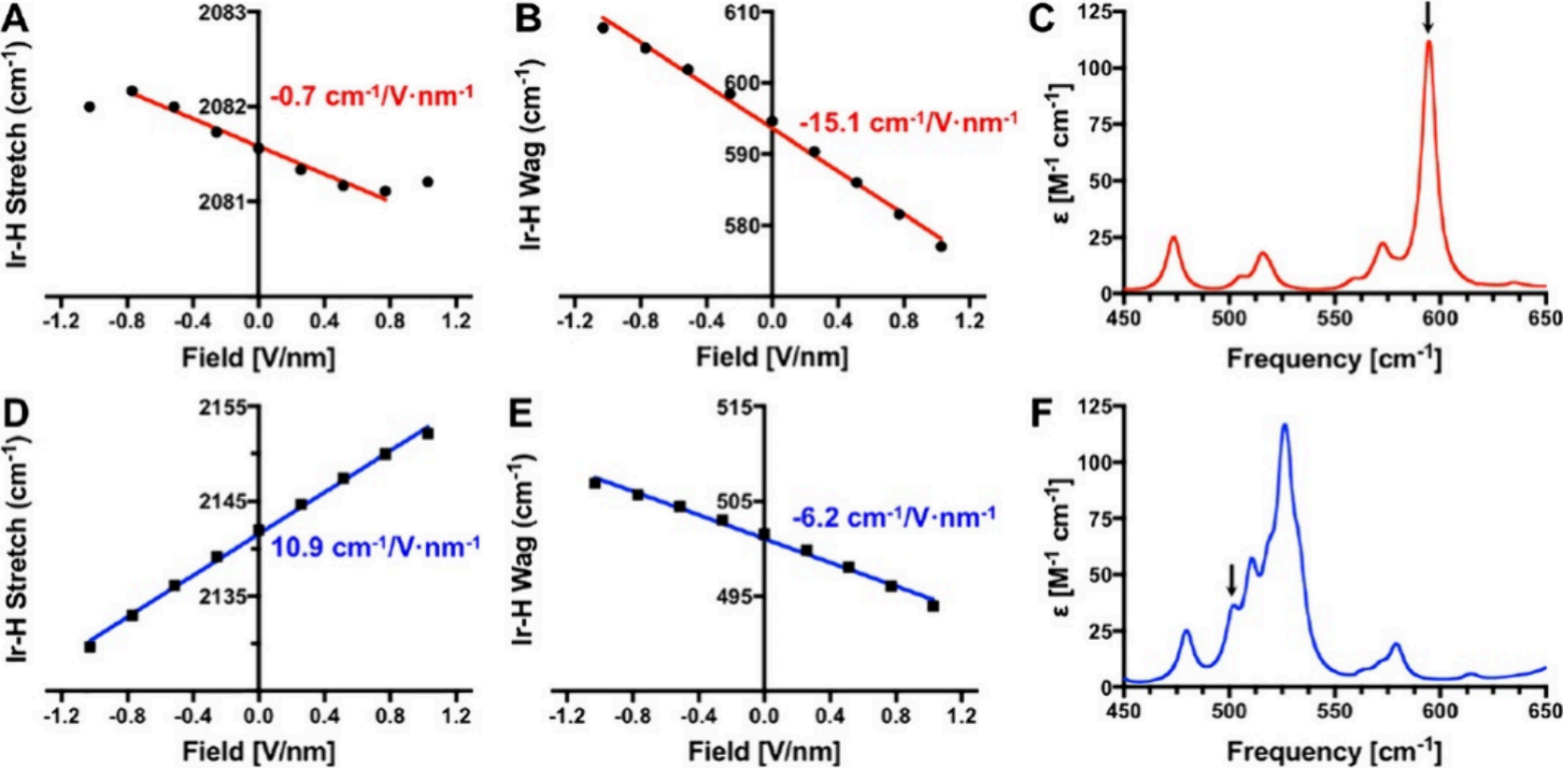
Field dependence of the Ir–H stretching frequencies of (A) [Ir-bpy-H]^+^ and (D) Ir-ppy-H and the Ir–H wagging frequencies of (B) [Ir-bpy-H]^+^ and (E) Ir-ppy-H from density functional theory calculations. Stark slopes from linear fitting are indicated in each plot. Computed IR spectra around the Ir–H wagging mode are shown for (C) [Ir-bpy-H]^+^ and (F) Ir-ppy-H. The relevant peaks are labeled with black arrows. Adapted with permission from ref (105). Copyright 2023 American Chemical Society.

Beyond conventional reporter molecules with −CN or −CO groups, a recent computational study by Kelly et al. proposed using the Ir–H bond vibrations when studying the effect of EF on hydricity (also see section 4.2), as this bond is cleaved and its strength is directly related to the spring constant that determines its vibrational frequency.^105^ In Figure 8A,D, the Ir–H stretching frequencies as a function of the applied field for the [Ir-bpy-H]^+^ and Ir-ppy-H are given, respectively.

While there is a significant shift in the Ir–H stretching mode in the case of the Ir-ppy-H complex (Figure 8D), [Ir-bpy-H]^+^ displays a much smaller dependence of the Ir–H stretching frequency on the EF with a slope of only −0.7 cm^–1^/V*nm^–1^ (Figure 8A). This means that the Ir–H stretch in the Ir-ppy-H complex can be used as a Stark probe but not in the [Ir-bpy-H]^+^ case. The authors therefore investigated the Ir–H wagging as a possible alternate vibration that should be affected by the changing field and electron density on the Ir metal center and hydride ligand. Here, they found that Ir–H wagging has a high shift of −15.1 cm^–1^/V*nm^–1^ for the [Ir-bpy-H]^+^ complex (Figure 8B), much more than the Ir–H stretching mode. Furthermore, the wagging mode is well separated from other modes in the spectrum, with a frequency of 595 cm^–1^ without applied filed (Figure 8C), making it a promising candidate to be used as a Stark probe. The field-dependent frequency shift of the Ir–H wagging in the Ir-ppy-H complex is lower than both its shift of the Ir–H stretching and the corresponding wagging in the [Ir-bpy-H]^+^ complex as well (Figure 8E). Still, with a slope of −6.2 cm^–1^/V*nm^–1^, this shift is significant. However, since the Ir–H wagging mode is in a crowded region of the complex’s vibrational spectrum, it is unsuitable to be used as a probe for EFs. Therefore, the [Ir-bpy-H]^+^ complex can be used as a probe for EF strength using the vibrational frequency shift of its Ir–H wagging mode, while the Ir-ppy-H molecules can be used similarly when observing the Ir–H stretching mode.

In the plasmonic enhancement field, a vibrational Stark effect with SAM probes is also applied to quantify the EF within the hot spots^106,107^ and detect the actual EF environment within the hot spots during catalysis.^13^ Notably, in a plasmonic enhancement field, there coexists a static direct current (DC) EF from the EDL and an electromagnetic alternating (AC) field at the surface. The effect on the vibrational Stark probes should be directly related to the DC field. However, studies using vibrational Stark probes based on SAMs with −CN groups have shown that on plasmonic metal surfaces, the optical AC field can also induce an extra DC field (local EF) at the interface.^108−110^ It should be noted here that there may be some influence on static electric fields introduced by the probing techniques. In these surface-enhancement-based techniques, the extent to which the addition of the plasmonic structures perturbs the EDL and further affects the characterization results remains debated. For instance, *in situ* shell isolated nanoparticle enhanced raman spectroscopy (SHINERS),^111^ employing shell-isolated plasmonic nanoparticles has been widely utilized in probing electrochemical electrode/electrolyte interfaces and identifying key intermediates in energy conversion systems.^112−115^ It was recently reported that the absolute frequency of certain modes of molecules are also affected and show a NP-distance dependence when the Raman signal is enhanced via the electromagnetic field enhancement mechanism in a SHINERS system, which indicates the additional NPs’ influence on the surface static electric field.^107^ When utilizing a scanning-tip-enhanced Raman experiment, other studies^116,117^ also reported substantial perturbation of the EDL in the presence of the STM tip, highlighting the potential sensitivity of the interfacial environment to physical and chemical modifications. SERS with nanoparticle-on-mirror (NPoM) scheme^13,23,118−121^ presents a similar controversial question on whether the probed IEF is affected. It has been reported^13,23^ that the molecules inside the “sandwich” structure show a Stark tuning slope similar to that measured by conventional SERS without sandwich structures, indicating the unaffected static IEF in the EDL; conversely, other studies^108,109,122^ claimed that the vibrational molecular probes are affected by the local electric field environment in a gap mode SERS, which can be modulated by the gap size and affected by the polarized NPs from the electron tunneling across the junction.

Recent advancements in utilizing vibrational Stark spectroscopic tools have also focused on directly measuring the EF on semiconductor-based photoelectrodes, demonstrating a new approach of generating EFs with photovoltages for chemical processes instead of applied potentials at electrolyte–electrode interfaces.^123,124^ In those studies, a 4-MBN SAM is used as a local EF reporter attached at the metal or semiconductor substrate. The studies presented in this section narrow the gap between heterogeneous and homogeneous catalysis, enhancing our understanding of how an electrode interface affects the electronic structure and reactivity of surface-bound species and how to utilize existing knowledge of the effects of inductive substituent groups on catalysis and other processes (discussed further in section 4).

## 3 Computational Methods for Including Electric Fields at Electrode Interfaces

### 3.1 Constant Electric Fields

Computational chemistry methods, such as DFT and MD are powerful tools for exploring microscopic processes at the atomistic level. In these systems, electrodes are modeled as clusters or periodic slabs consisting of several layers of atoms and the EF is perpendicular to the slab/cluster surface. To investigate the effect of applied EF on these systems, a straightforward and widely utilized approach is by directly applying a constant EF. For cluster systems, this approach is relatively simple: it simply adds an electric potential energy term (*V*_E_) to the Hamiltonian for each particle:
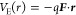
4Here, *q* is the charge of the particle, ***r*** its coordinates, and ***F*** the electric field. This introduces an additional force *q**F*** acting on each particle equal to the gradient of this field-induced potential energy term. The electric field to be applied in computational simulations can be estimated by the experimentally applied potential φ and the potential of zero charge (PZC, *φ*_PZC_):
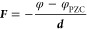
5

Here, ***d*** is the approximated double layer thickness. When an electric field is introduced, however, periodic boundary conditions create discontinuities in the electrostatic potentials generated by EFs. This issue is addressed through the inclusion of a planar dipole layer. This dipole correction method is originally developed to counteract the artificial electric field produced by asymmetric slabs with net dipoles and then generalized to applied external EFs.^125,126^ While *in silico* results obtained through applying static EFs can be challenging to compare to experimental results, where the EF is induced through applied electrochemical potentials resulting in effective EFs that are challenging to determine, it is widely applied due to its relative simplicity and capturing of most of the fundamental effects of EFs on surface-molecule systems.^23,91,105,127−135^

### 3.2 Varying Electron Counts on the Electrode

A more elaborate inclusion of the EF is to explicitly implement charging and discharging processes by adding or removing electrons to the electrode. The EF is then implicitly induced through changes in charges and their distribution. One immediate difficulty in periodic systems is maintaining charge neutrality to prevent divergence in the electrostatic energy. The charge neutrality can be achieved by introducing uniform background counter charges.^103,136−140^ In this approach, the potential of the electrode with the added charge can be calculated as the bulk solvent electrostatic potential minus the Fermi-level-associated potential, and should be referenced to the PZC.^141,142^ The counter charges can also be planar charge sheets,^142−145^ explicit counterions,^146,147^ or an oppositely charged slab.^148^ Distribution of these nonuniform counter charges should be chosen to represent the electrochemical double layer. Varying the count of electrons in the system more closely captures the origin of the EF from an applied electrochemical potential. Nevertheless, this approach still requires careful evaluation of the number of electrons and the distribution of the counter charge, as the experimental EFs depend on many factors such as electrolyte and surface morphology that are challenging to capture in model systems.

### 3.3 Grand Canonical Ensemble Approaches at Constant Electrode Potentials

Experiments are mostly carried out under constant potential conditions, while the number of electrons on the electrode varies. In practice, the field strength of the applied constant EF, or the number of added/removed electrons to/from the electrode, should be scanned to reproduce the experimentally applied potential or experiment observations. A more faithful simulation of an electrode process should be in the grand canonical (GC) ensemble. The grand potential Ω depends on the chemical potential μ (corresponding to the electrode potential), volume *V* and temperature *T* and is defined as
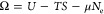
6where *U* is the internal energy, *S* the entropy, and *N*_e_ the number of electrons in the system. At constant grand potential Ω, a change in chemical potential μ, therefore, requires a change in the number of electrons *N*_e_. In GC methods, the number of electrons and the electron wave functions/densities are optimized together.^141,149−151^ For example, in the grand canonical self-consistent field (GC-SCF) approach, the Fermi energy at *j*th self-consistent field iteration (*E*_F_^*j*^), up to which the Kohn–Sham orbital should be filled, is a linear combination of the targeted Fermi energy (*E*_F_^tgt^, decided by the potential) and the Fermi energy of the previous step (*E*_F_^*j*–1^), i.e.,
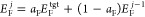
7where *a*_F_ is the combination coefficient.^149,150^ While it is computationally extremely costly, the induced field in this method is the most closely related to the experimental reality, where a constant potential is applied.

## 4 Controlling Reactivity with Electric Field

Development of the vibrational Stark effect spectroscopies and probes has equipped us with the knowledge of interfacial environments and characterization methods of local EFs. The effects of EF on surface bound substrates and catalysts are now under investigation. How an EF affects the reactivity of these species at electrode surfaces is mostly attributed to two mechanisms: (1) EF facilitations or prohibitions of electron or proton transfers, and (2) EF interactions with molecular dipoles and polarizabilities that change the energetics and properties of substrates or catalysts.

### 4.1 Electric Field Effects on Transfer of Charged Particles

EF effects have been widely studied in many electrochemical processes with interfacial proton and/or electron transfer reactions.^87,132,152−155^ Conventional electrochemical methods (impedance, voltammetry, etc.)^156−158^ have been used to study the proton transfer processes between acid/base species in the double layer, but it is crucial to provide molecular-level insights into these EF-driven/affected proton transfer processes. Herein, we present a brief review of progress in utilizing vibrational spectroscopic tools for studying the EF effects on interfacial proton transfer and chemical reactions.

Most local EF reporters described above are SAMs containing nitrile or carboxyl groups due to their intense vibrational spectroscopic signals. This strategy is also employed in studies of EF-driven/affected proton transfer reactions. In these systems, electrodes are functionalized by SAMs with pH-sensitive and IR/Raman-active functional groups. The local interfacial environment can affect population distributions of protonated and deprotonated species at interfaces and create changes in the spectra.^159−161^ Thus, by analyzing the relative peak intensity/amplitude changes of deprotonated/protonated species as a function of potential, the interfacial proton-transfer-related physicochemical parameters, such as the acid dissociation constant (p*K*_a_), can be obtained directly.

Early studies applied SERS to probe local proton transfer reactions at different electrochemical potentials and study their potential-dependent interfacial p*K*_a_ values compared to values in bulk solution. For example, Cao used 2-aminoethanethiol (2AT) SAMs on Au electrodes as a SERS substrate to study the potential-dependent interfacial proton transfer reaction of an amino group.^162^ The author found that the surface p*K*_a_ of the amino group is controllable under different biases, where the p*K*_a_ values are 5.0 ± 0.2 (0 V), 4.2 ± 0.2 (0.1 V), and 3.4 ± 0.2 (0.2 V) (vs Ag/AgCl). Ma et al. obtained a similar result using 2-mercaptobenzoic acid (2-MBA) immobilized on a Ag electrode for the SERS study.^163^ By analyzing the vibrational modes of COO^–^ and C-COOH, the authors found that the applied potential created a significant difference between the surface p*K*_a_ and bulk solution p*K*_a_ of 2-MBA. The authors proposed that this arises from changes in the activity of protons at the interface and changes in the electron density governed by the applied potential within the EDL. As the EF effects are through-space for SAMs of nonconjugated molecules, there should be a distance dependence for proton transfer reactions. This was observed in one study showing chain length-dependent p*K*_a_ changes of alkanethiols with terminated −NH_2_ groups, using impedance-based titration techniques and SERS to characterize the degree of surface order.^164^

A more in-depth understanding of the thermodynamics of the interfacial proton transfer process was later reported by Ge et al., who studied 4-mercaptobenzoic acid (4-MBA) on Au with VSFGS and electronic-structure calculations.^165^ Experimental results again indicated a potential-dependent interfacial p*K*_a_ change. However, the authors went beyond the conventional interpretation in which the deprotonated charged species and protonated neutral species interacted differently with the EF. Their interpretation claimed that the electrode-adsorbate could be regarded as a delocalized system, and the electron was not necessarily an integer. The authors finally united the two interpretations with a simplified, purely electrostatic model and performed calculations for the previously studied 2-MBA on Ag system to verify its applicability.

**Figure 9:**
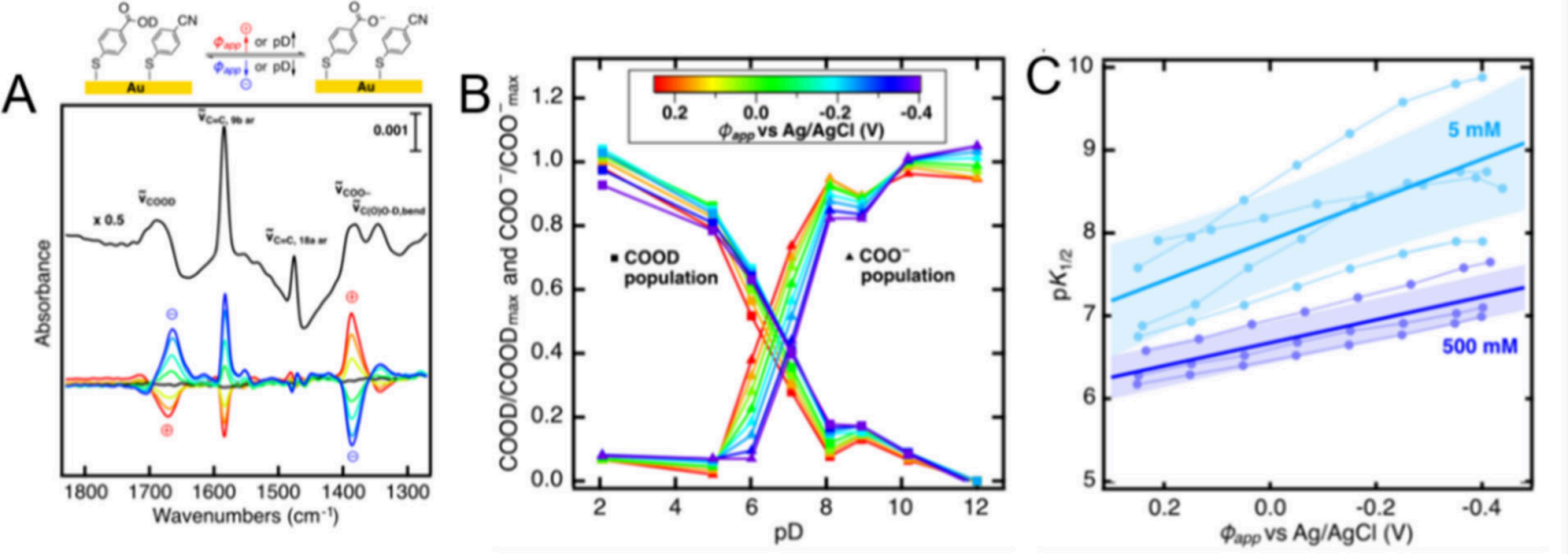
(A) Represented SEIRAS spectrum of (de)protonation reaction of 4-MBA at the interface and corresponding difference SEIRAS spectra from 0.3 to −0.4 V vs the spectrum at −0.05 V. (B) Surface population of COOD and COO^–^ under different pD buffer conditions and different potential conditions. (C) Plot of p*K*_1/2_ vs potential. Adapted with permission from ref (29). Copyright 2021 American Chemical Society.

The EF-affected interfacial acid/base equilibrium was systematically studied in two more recent works by Delley et al., which reported the concurrent interfacial EF and acidity by leveraging a mixed SAM method with 4-MBA as the local effective acidity reporter and 4-MBN as the local EF reporter probed by *in situ* SEIRAS (Figure 9).^29,166^ The authors systematically investigated how the population of the interfacial acid/base form changes as a function of the potential, bulk pH (pD), electrolyte concentration, and electrolyte cation types in tandem with local interfacial EF monitoring. They also found the interfacial effective acidity (p*K*_1/2_) changes as a function of potential, and the EF reporter reflects the majority of potential drop (∼90%) occurs between the electrode and SAM. By changing the electrolyte content on ionic strength and cation types, the authors also highlighted the specific interactions (and penetration) of ions with the SAMs, which can be the key parameter in fine-tuning the surface electric potential profile and the surface acidity.

As an example of the EF effect on electron charge transfer, external EF control of the Diels–Alder (DA) reaction was proposed by the computational work of Meir et al.^167^ EF antiparallel to the direction of electron flow lowered the activation barrier and accelerated the reaction. Increasing the EF strength to a critical value resulted in a stepwise mechanism via a zwitterionic intermediate. The effect of acceleration was later verified by experimental work using the scanning tunnelling microscopy break-junction approach (STM-BJ).^168^ A rigid norbornylogous bridge with a terminal double was attached to a flat gold with a well-defined orientation, reacting with a furan derivative attached to the STM gold tip. In this model system, an external EF facilitates electron flow to achieve a 5-fold increase of reaction rate, as measured by the frequency of single-molecule junction formation.

### 4.2 Electric Field Effects on Immobilized Substrate/Catalyst Properties

EF effects on a molecule’s potential energy *V* can be written as
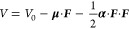
8where *U*_0_ is the zero-field potential energy, ***F*** is the EF vector, **μ** is the molecule’s dipole moment, ***a*** is the polarizability tensor, and where field inhomogeneities and higher-order polarizabilities are neglected.^169,170^**α**•***F*** can be regarded as the EF induced dipole moment. Therefore, the EF can directly alter the molecular energy and change the relative stabilities of different transition states or products, resulting in different product distributions. On example is the aforementioned computational study of the Diels–Alder reaction.^167^ When the EF is perpendicular to the electron flow, different alignments between the EF and the alkene (maleic anhydride) dipole (parallel or antiparallel) predict different endo/exo reaction barrier heights and therefore different product selectivity. Experimentally, it has been shown that EF can tune the *cis*-stilbene oxide rearrangement at an Al_2_O_3_–solution interface.^171^ The aldehyde to ketone product ratio changes from 1:3.7 to 16.9:1 (about 63-fold) with the increasing EDL charge density.

This field control of product distribution is of great interest in the design of molecular devices and molecular switches. For example, azobenzene can undergo *cis–trans* isomerization activated by photoexcitation in solution/gas phase or resonant/inelastic tunneling of electrons when absorbed on a surface. EF induced *cis–trans* isomerization was investigated and observed in the STM junction for 3,3′,5,5′-tetra-*tert*-butylazobenzene on a Au(111) surface.^172^ DFT computations showed that this is achieved by bias-induced EFs affecting the ground state potential energy surface with contributions from both dipole and polarizability terms.^173^ In another work, a *p*-((8-hydroxyquinoline)azo)-benzenethiol (SHQ) monolayer was immobilized on a silver electrode.^174^ SERS characterization revealed that the keto-hydrazone tautomeric form of SHQ was favored under a negative electric potential, while the enol-azo tautomer was favored with a more positive potential. DFT calculation suggested a large dipole difference between the two tautomeric forms, explaining this potential-dependent behavior as the EF-dipole interaction. As the enol-azo form possesses much higher metal ion (Cu^2+^) binding affinity, the authors also demonstrated that the Cu^2+^ binding equilibrium was also controlled by the potential, which provides a strategy for designing electrically switchable molecular devices for metal ion complexation.

**Figure 10:**
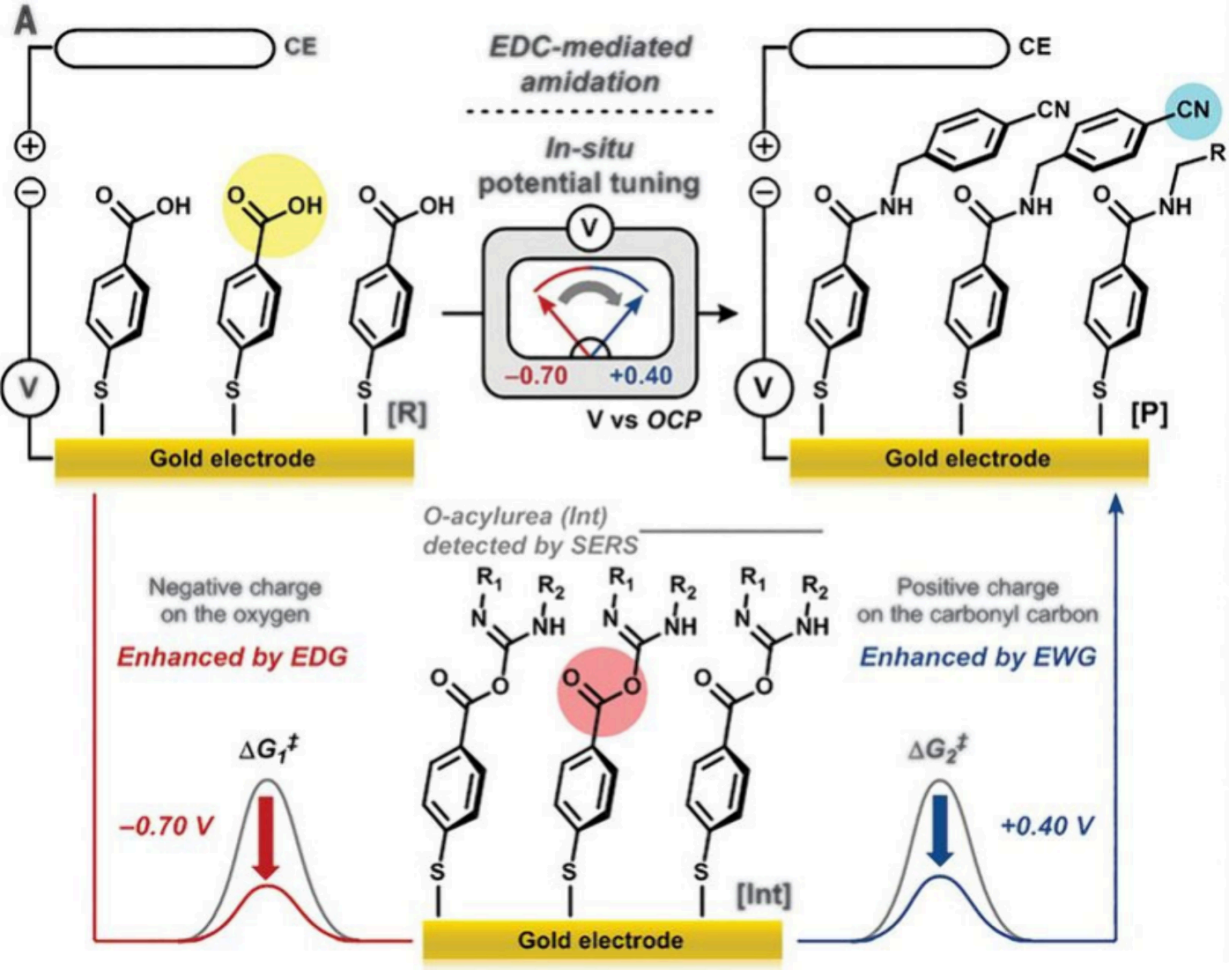
Two-step amidation of surface-immobilized 4-mercaptobenzoic acid. The first step from benzoic acid to *O*-acylurea was assisted by application of a negative bias (red). The subsequent reaction from *O*-acylurea to benzonitrile was aided by a positive potential (blue). Adapted with permission from ref (15). Copyright 2020 American Association for the Advancement of Science.

In many other studies, the polarization induced by the applied EFs is usually is referred to as the electro-inductive effect, when explaining the potential origin of frequency shifts of the adsorbates other than the Stark effect.^101^ As described in section 2.5, it was shown by Sarkar et al.^21^ that the Stark shift of surface-bound benzonitrile molecules were correlated to the Hammett parameter, a quantitative measure of a group’s electron withdrawing capability, or inductive effect. The electro-inductive effect due to an applied bias on the reactivity of surface-bound reagents has been probed using SERS on gold-bound species with three known reactions: a base-catalyzed ester hydrolysis, a Suzuki–Miyaura cross-coupling, and a two-step carboxylic acid amidation.^15^ The first study monitored the saponification of gold-bound *tert*-butyl 4-mercaptobenzoate to 4-mercaptobenzoate at different biases. Relative SERS intensities of *v*(C=O) in the reactant and *v*(COO^–^) of the product showed that, as expected, positive potentials accelerated the reaction, while negative voltages almost completely inhibited product formation. In the second study, the rate of the Pd-catalyzed cross-coupling of surface-bound 4-bromobenzenethiol and solution-state phenylboronic acid was monitored by the disappearance of the *v*(C–Br) stretch from the reactant and the appearance of new *v*(C–C)_ring_ stretches from the product, as well as time-of-flight secondary ion mass spectrometry (ToF-SIMS). Predictably, the reaction rate increased with increasingly negative potentials, as the applied bias mimics substitution with electron-donating groups known to increase reactivity. The third study utilized a known two-step reaction where the energetic barrier of the first step is lowered by increasing the nucleophilicity of the parent compound and the second step is favored by an increase in electrophilicity. Using a monolayer of 4-mercaptobenzoic acid, the first of the two-step carbodiimide-mediated amidations (from benzoic acid to *O*-acylurea) was assisted by a negative bias of 0.70 V, and the second step (from *O*-acylurea to benzonitrile) was assisted by a 0.40 V positive potential (Figure 10). Fractional charge density functional theory (FC-DFT) has been used to investigate the Stark shift of 4-MBN,^21^ and the base-catalyzed saponification reaction.^15^ The computational results support an apparent inductive electronic effect on surface-bound species.^102^

A recent computational study has dissected the influence of EFs of varying strength and orientation on the hydride transfer model reactions:
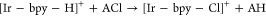
9
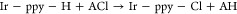
10with iridium complexes adsorbed onto a Au(111) surface.^105^ DFT calculations of reaction free energies of the two model reactions determined the tendency for the complexes to donate a hydride to an acceptor (hydricity) while applying EFs of varying strengths.

**Figure 11:**
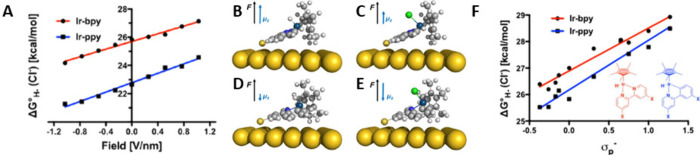
(A) Hydricities of complexes [Ir-bpy-H]^+^ and Ir-ppy-H as a function of the applied EF with best-fit lines of +1.4 and +1.7 kcal mol^–1^/V nm^–1^, respectively (*R*^2^ > 0.98 for both fits). Minimum energy configurations of Ir complexes on Au, including (B) [Ir-bpy-H]^+^, (C) [Ir-bpy-Cl]^+^, (D) Ir-ppy-H, and (E) Ir-ppy-Cl. (F) Effective hydricity values of 4,4′-substituted Ir-bpy and Ir-ppy complexes as a function of the Hammett parameter (σ_p_^–^). Adapted with permission from ref (105). Copyright 2023 American Chemical Society.

As shown in Figure 11, the hydricities of both complexes, [Ir-bpy-H]^+^ and Ir-ppy-H, shift as a function of applied EF in the range of ±1 Vnm^–1^. Between these two extremes, the hydricities of the complexes shift by approximately 3 kcal/mol, with negative bias facilitating and positive potentials inhibiting the hydride transfer. Interestingly, the authors found that applying a positive field lowered the free energy of both the [Ir-bpy-H]^+^ as well as [Ir-bpy-Cl]^+^, but increased the free energy of the corresponding Ir-ppy-H and Ir-ppy-Cl. This effect could be explained by the molecular dipole moment orientation of the Ir-bpy and Ir-ppy species: while the dipole moment of the former (Figure 11B) has a strong component pointing along the surface normal away from the surface and parallel to the field, the latter has a dipole moment orientation directed toward the surface (Figure 11C), opposite to the applied field. Even though the stabilization differs between the complexes, the relative shift from the hydride complex to the respective chloride is constant in both the bpy and ppy iridium complexes, as visible in Figure 11D,E. While the absolute energy of the Ir-bpy and Ir-ppy complexes was therefore affected inversely due to their differently aligned dipole moments, the relative shift between hydride and chloride complexes remained similar.

The suggested changes in hydricity upon applying an EF are quite high and on the order of changes induced by chemical substituents of varying electron withdrawing and donating character. As a comparison of the hydricity change induced by chemical substituents and EFs, the authors expanded the study to include a total of 10 Ir-bpy and 10 Ir-ppy complexes substituted on the 4 and 4′ positions by chemical substituents. A wide range of electron donating/withdrawing characters were employed, as specified by Hammett parameters ranging from the electron donating OH group (−0.37) to the heavily electron withdrawing NO_2_ (1.27). In Figure 11A, the relation between Hammett parameter and hydricity is linear for both complexes (*m* = 1.6 kcal/mol, *R*^2^ = 0.96 for Ir-bpy; *m* = 1.9 kcal/mol, *R*^2^ = 0.94 for Ir-ppy), with total shifts of 2.7 kcal/mol for the Ir-bpy family and 3.0 kcal/mol for the Ir-ppy complexes, fairly close to the 2.9 and 3.3 kcal/mol shifts for the [Ir-bpy-H]^+^ and Ir-ppy-H between −1 V/nm^–1^ and +1 V/nm^–1^, respectively. This clearly shows that applying EFs can affect hydricity in the same order of magnitude as introduction of chemical substituents.

### 4.3 Carbonaceous Electrode Conjugation and Position in the Double Layer

**Figure 12:**
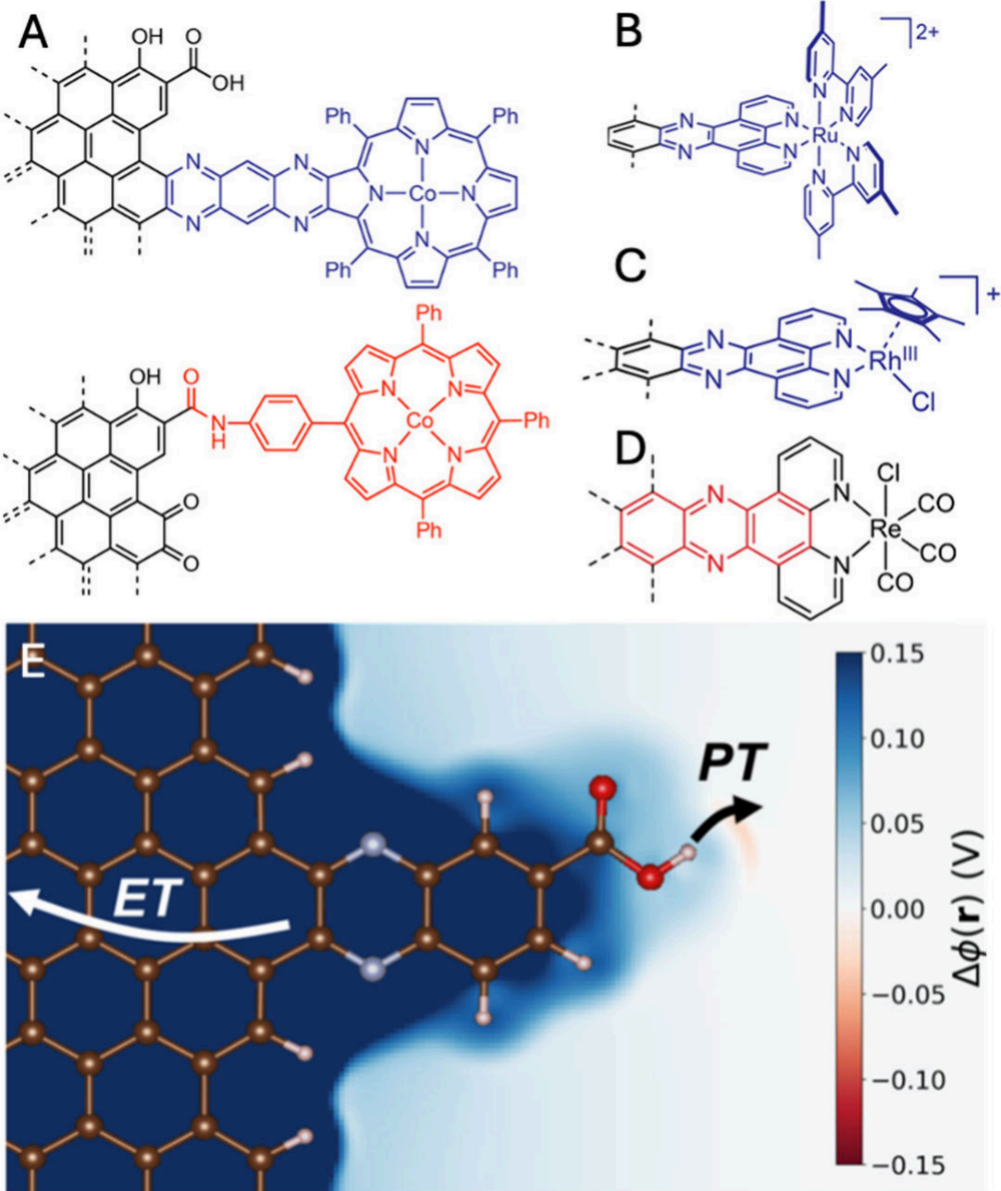
(A–D) Illustrations of Co, Ru, Rh, and Re catalysts attached to graphitic electrodes by the Surendranath group. (E) Scheme of the potential driven proton-coupled electron transfer (PCET) reaction at the graphite-conjugated catalysts (GCCs). (A–C) Adapted with permission from ref (180). Copyright 2019 American Chemical Society. (D) Adapted with permission from ref (176). Copyright 2016 American Chemical Society. (E) Adapted with permission from ref (155). Copyright 2020 American Chemical Society.

In a departure from more conventional surface functionalization chemistries, Surendranath and co-workers have focused on developing highly conjugated and proximal attachment schemes to carbonaceous electrode surfaces. They have demonstrated that the reaction of *O*-quinone moieties on oxidized graphitic surfaces with 1,2-diaminobenzene or related species proceeds rapidly to form pyrazine moieties on the surface.^175^ This chemistry can be used to introduce catalytic functionality to graphitic surfaces. A noteworthy example is when a Re(5,6-diamino-1,10-phenanthroline)(CO)_3_Cl complex is used in the surface reaction, they found that it formed an atomically precise metal binding site which introduced CO_2_ reduction activity to the otherwise inert surface.^176^ Compared to more traditional attachment motifs, this system showed no detectable evidence of Faradaic redox features of the Re center at the surface, as would be expected from the behavior of homogeneous rhenium phenanthroline analogues. Under conditions suitable for CO_2_ reduction, the catalytic activity for electrochemical CO_2_ reduction of the homogeneous analogue Re(phen)(CO)_3_Cl plateaus at potentials cathodic of the Re^+1/0^ redox couple, corresponding to the limited turnover frequency (TOF) of the homogeneous catalyst. Deviating from this behavior, the rhenium modified graphic electrodes described in this study show a continuous increase in catalytic turnover rates with respect to the cathodic bias. This suggested that the reactivity of the surface-bound active site is changing as a function of electrode bias. Surendranath has since expanded the set of graphite-conjugated transition metal complexes which exhibit this behavior, including rhodium pianostool complexes,^177^ ruthenium polypyridines^177^ and cobalt porphyrins^178^ (Figure 12A–D).

This behavior has been explained as a result of the complete conjugation of the electronic states of the electrode and the surface bound molecules.^177^ The description invoked by Surendranath is that the redox active metal is placed in such close proximity to the electrode surface that the active site is spatially located within the electrochemical double-layer. Because there is no distinction between the catalyst and the bulk electrode, no Faradaic charge transfer events are observed. However, chemical steps can still be observed electrochemically as long as the steps involve the passage of charge across the double layer, such as the ejection of anionic ligands or proton transfer.

The potential-dependent change in maximum TOF of the graphite-conjugated catalysts is evidence that the reactivity of those surface-bound active sites is most similar to heterogeneous electrocatalysts (e.g., metal surfaces). In drawing parallels to heterogeneous electrocatalysts, Surendranath also investigated the potential-dependent reactivity of surface-hydride species on Ni, Pd, Pt, and Au electrodes, and found substantial influence of electrode bias on hydride reactivity.^179^ In both graphite-conjugated and heterogeneous systems shown in Figure 12, Surendranath and co-workers found this observable potential-dependent influence on surface bound reactivity. The theoretical origin of these effects has been elusive to thoroughly describe, as both electronic conjugation and interfacial EFs provide plausible mechanisms for the observed bias-dependent reactivity.

The most direct probe of this question comes from Surendranath’s study of cobalt tetraphenylporphyrin (CoTPP) bound by a longer aliphatic linkage to glassy carbon electrodes in aqueous and organic media.^178^ In aqueous media, no redox features are observed in CV and the molecule again acts most like a metal surface in catalyzing the hydrogen evolution reaction (HER); the potential drop exists between the electrode-coupled cobalt center and solution, consistent with previous results and suggestive of the Co active site residing within the double layer. However, in an organic solvent, molecular redox features are revealed, suggestive of outer sphere electron transfer to the metal center and redox mediation under catalytic conditions. The solvent-dependent position of the metal center with respect to the double layer greatly affects the catalytic process toward HER in each system, and the position in the double layer is reminiscent of a previously discussed study which cited hydrophobic interactions as the cause for deviations from the typical double layer structure.^64^ Further spectroscopic probing of these systems would be extremely useful to map interfacial electric fields and the structure of the double layer in different solvents, but these studies are challenging due to the absorbing nature of the carbonaceous supports.

## 5 Magnetic Fields for Chemical Processes

EFs are a function of the spatial distribution of charge density. Electrochemical reactions, however, include the transfer of charge, leading to fluctuations of the charge density in time. Such changes induce magnetic fields (MFs), which are intrinsically connected to EFs through the relationships formalized by Maxwell’s equations. The effects of MFs in catalysis are known in the literature and are often used in tandem with electric fields as an additional tunable effect to enhance selectivity or alleviate the costs of electrochemical overpotentials. For physical processes, MF effects include magnetohydrodynamic effects that enable mass transport of ions to/from the electrode surfaces^181−183^ and magnetothermal effects that permit heating localized at reaction interfaces for kinetic enhancement without wasting energy heating up a solvent bath.^184−189^ By contrast, MF effects on chemical processes focus on manipulation of radical pairs and spin polarization enabling selection of desirable reaction pathways.^190−195^ In the sections that follow, we focus on the MF-facilitated chemical processes and present ways in which magnetic fields can bolster electrochemical processes in applications such as hydrogen or oxygen evolution reactions.

### 5.1 Magnetic Field Control of Chemical Pathways for Photochemistry and Redox Processes

**Figure 13:**
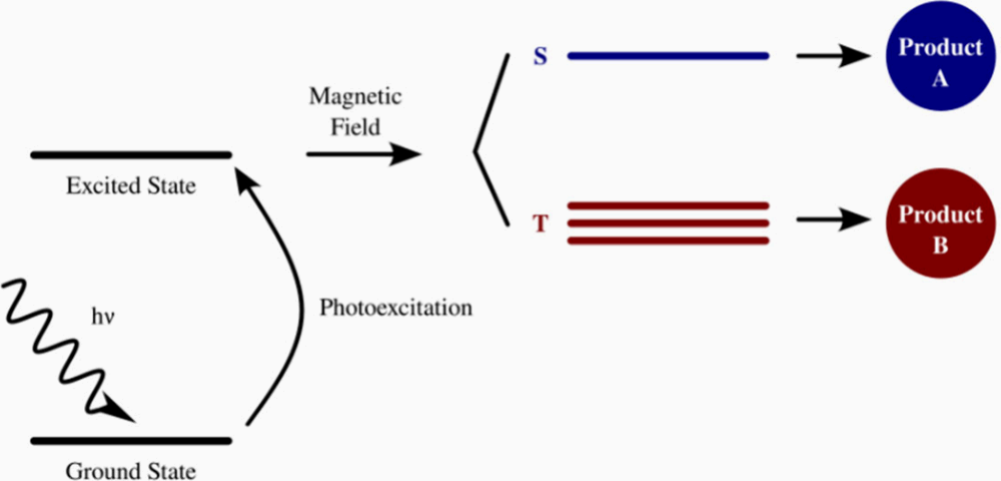
Example of prototypical photochemical reaction: a ground state molecule is photoexcited, and upon reaching the excited state, a magnetic field induces electronic state anisotropy. Depending on the strength and direction of the magnetic field, this may favor reactivity along singlet (product A) or triple pathways (product B).

One of the earlier experiments showcasing the usage of magnetic fields to control chemical properties was demonstrated by Blankenship and co-workers.^196^ In this experiment, the accessible electronic states of the bacteriochlorophyll P-870 protein of Rhodopseudomonas spheroids were manipulated using magnetic fields. As illustrated in Figure 13, photoexcitation of the P-870 complex was proposed to generate a radical pair system through photoelectrochemical electron transfer to an acceptor. The recombination of these charges can lead to either a singlet or triplet state of P-870. In the absence of a magnetic field, the triplet to singlet quantum yield is 75:25. Application of a 100 mT magnetic field lifts the triplet degeneracy, resulting in a quantum yield of 50:50 *T*/*S* (only the *T*_0_ state is degenerate with *S*_0_). This phenomenon has further implications in fluorescence processes and overall electron transfer kinetics, as was demonstrated in a follow-up study.^197^ Similarly, pathway selectivity by magnetic field has been demonstrated for photochemical conversion of anthraquinone reactants.^198,199^ Other studies also investigated reaction control based on the spin character of the available electronic states (single-triplet, triplet–triplet processes) under a range of magnetic fields for radical pair species.^200^

Recent experiments also make use of intramolecular magnetic properties to control radical pair generation for water oxidation.^201^ Zhang et al. propose a radical mechanism reaction for water oxidation with three competing pathways atop an Fe_3_O_4_ nanoparticle ferromagnetic substrate functionalized with chiral adsorbates:
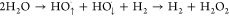
11
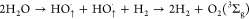
12
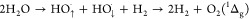
13

The generation of hydrogen peroxide is kinetically accessible compared to singlet oxygen generation. Triplet oxygen is about 1 eV more thermodynamically stable than singlet oxygen, but accessing this pathway requires ensuring the availability of a sufficient number of spin-aligned hydroxyl radicals. Ensuring that the generated radicals have the same spin character would potentially facilitate the triplet oxygen pathway alongside hydrogen evolution, henceforth termed the oxygen evolution reaction (OER).

Other studies examined both the dependence of electrochemical water splitting in the presence of magnetic fields and its relation to the magnetic properties of the catalytic substrate. The OER activity has been studied under a magnetic field of at most 371 mT at the anode interface and observed a maximum enhancement of 4.7%.^202^ An improvement of the catalytic activity of decorated nickel electrodes has been observed for water splitting by about 40% under magnetic fields of at most 450 mT compared to activity in the absence of applied magnetic field.^203^ Combined experimental and theoretical studies also showcase that the local electronic structure effect of the catalysts may confer local magnetic effect, leading to enhanced catalytic activity even in the absence of external magnetic field.^204,205^

Other reactions of environmental interest that may benefit from magnetic enhancement^206^ include: degradation of chemical waste products (phenol, methyl orange, Rhodamine B, Rose Bengal over TiO_2_;^207^ phenol red on Bi_1–*x*_R_*x*_FeO_3_, R = Ce/Tb, in varying stoichiometric ratios *x*;^208^ norfloxacin, enhanced from 34.7% in the absence of a magnetic field to 68.8% with a 200 mT field after 90 min^209^); HER;^210^ CO_2_RR;^211^ nitrogen fixation and reduction (42% enhancement compared to no magnetic field with BaTiO_3_).^212^ These are accelerated due to the presence and stabilization of radical species as intermediates that assist with chemical transformation. Thus, the design of magnetically susceptible/active catalysts and usage of applied external magnetic fields may provide a venue to control chemical reactivity in addition to EF control.

### 5.2 Magnetic Field Control of Spin-Dependent Electron Transfer Processes

**Figure 14:**
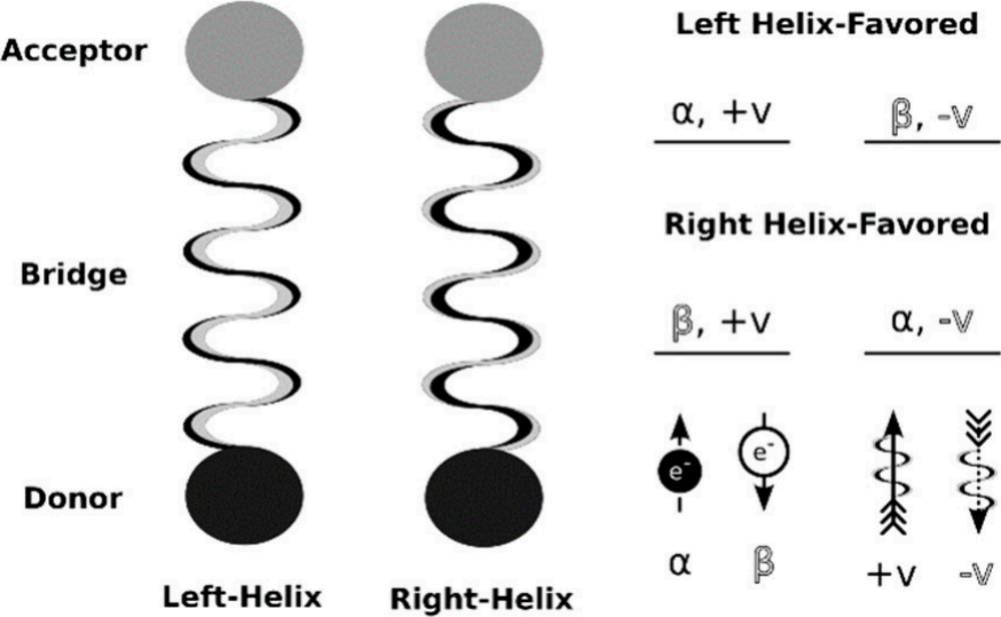
Prototypical schematic of the CISS effect where the chirality of the bridge affects the transport of electrons from the donor to the acceptor. One chirality enhances the transport of electrons with a particular spin state, increasing its transmission across bridge due to the favorable interactions with the chemical properties of the bridge. Its spin counterpart is hindered and thus observed in reduced percentage at the acceptor molecule.

One class of systems that has been under investigation for the last two decades is that responsible for chiral induced spin selectivity (CISS). These systems consist of donor–bridge–acceptor triads, where the donor segment transmits electrons across a bridge, and an excess of a particular electronic spin state is observed at the acceptor end (Figure 14). Typically, the bridge molecule has some global chiral properties, often due to its helical shape and sometimes due to point chirality (enantiomeric centers).^213,214^ Possible explanations of this phenomenon include the coherent motion of the electron through the chiral bridge electron density (akin to superexchange) or incoherent transfer based on sequential hopping, which may be influenced by spin–orbit coupling or overall induced magnetic field to the nonuniform chiral electron density along the bridge. Experimental design to isolate the exact mechanism is challenging due to the inability to continuously tune the possibly relevant chemical parameters.^215^

The CISS phenomenon is more pronounced upon application of an external magnetic field that enhances the effect of the molecular ensemble. For example, a recent report described a MF-dependent anisotropic tunnelling current measured in a 2D TaS_2_ atomic crystal material functionalized with R- or S-α-methylbenzylamine, with a reported spin polarization ratio of 60%.^216^ Another example demonstrates the CISS effect in a molecular triad, where the authors present the coherent mixing of the spin eigenstates of the system as being responsible for the spin enhancement phenomena.^217^ These effects of spin-control by MFs could enable more careful control of photochemical processes as well as manipulation of quantum information, as required by quantum devices.

## 6 Conclusions and Perspectives

Nature creates and implements local EFs as necessary components of biological functions in membranes and enzymes. The formation and utilization of tailored local EF environments at electrode interfaces represent a promising area of study toward materials fabrication for electron transfer and catalysis.

Initial probes of the EDL focused on the vibrational Stark shifts of the adsorbed molecules with strong vibrational handles. Further efforts to characterize the interfacial EF used the vibrational Stark shifts of SAMs monitored by surface-specific techniques such as SERS, SEIRAS, and VSFGS, as well as computational methods. These studies probed the EDL and helped to validate the Gouy–Chapman and Guoy–Chapman–Stern models, providing a valuable jumping-off point toward modeling more complicated systems. Interrogating these systems has revealed a complex interaction of influences, including and beyond descriptions by mean-field models, such as field inhomogeneity, electronic vibrational effects, inductive effects, and solvent/electrolyte effects.

This characterization of the interfacial environment may inform system design toward control over the reactivity of surface-bound species. Work discussed in section 4 extends the use of well-understood inductive effects of functional group substitution on molecular catalysts to heterogenized systems. Computational studies show that through immobilization to a biased electrode, the electronic structure of surface-bound species, including catalysts, may be altered by the electro-inductive effect, allowing the rate and selectivity of chemical processes to be optimized. Similarly, MFs may be employed to enhance processes like electron transfer and catalysis in their vicinity. While the studies discussed in the review have built a foundation for employing external EFs and MFs to manipulate interfacial processes at electrode surfaces, challenges remain toward practical applications. Critical aspects to achieve this purpose include:

- *Surface-attachment strategy and control of orientations*. Conventional surface attachment on spectroscopically accessible surfaces such as gold are thiols and isocyanides, which have a limited potential window for stability. Stronger attachments to electrode surfaces which can weather catalytic conditions are necessary to study and utilize electro-inductive effects on catalysis. As the molecule interacts with the EF through the permenent and induced dipole moment, it is also critical to control the orientation of the suface-attached molecule for the optimal alignment of the dipole moment with the applied EF. Ideally, a thorough study of this aspect will enable a modular design of EF-responsive ligands for electrode functionalization.
- *EF effects on elementary catalytic steps: direction, activity, and selectivity*. EF effects have been studied for elementary reaction/catalysis steps like PCET and hydride transfer (hydricity). Investigations into the effects of electrode immobilization on other catalytically relevant elementary steps are now warranted. For example, oxidative addition is favored by an electron-rich metal center, while reductive elimination is aided by electron deficiency. Conventional catalyst design via substituent modification aims to balance the electronic character of the active site to favor both elementary steps, often a damaging compromise for each individual step. In a heterogenized system, EFs can be introduced and switched to control the reaction direction *in situ*, enabling catalytic reactions otherwise difficult to achieve and/or catalyst design focused on other aspects like TOF, selectivity, etc. Also, as the EF interaction with different species involved in the reaction process changes their relative energy, it can be utilized to enhance reactant/catalyst activity and promote/alternate product selectivity.
- *Experimental and computational method development*. Developing new experimental and computational methods can help extend the frontier of utilizing and understanding EF effects. From the experimental perspective, more investigations are needed to validate or refine electric field models across more complicated but universal scenarios. Challenges remain in understanding the electric field inhomogeneity and distributions, and their ruling factors in cases such as solid–liquid–gas three-phase interfaces, interfaces involving the repulsion or restriction the diffusion of certain species (i.e., membrane-electrode assemblies). The development of new *in situ* spectroscopic tools, such as multidimensional surface sensitive/selective vibrational spectroscopy, is another promising direction. On the computational side, current studies of chemical reaction at the electrode interface are mostly DFT calculations on static geometry-optimized structures, while MD simulations are mainly performed with classical force fields and focus on EDL structures and its molecular details. To include dynamical descriptions of chemical processes in the electrode interface system, ab initio molecular dynamics (AIMD) should be utilized to accurately describe the bond breaking/forming processes and polarized environments due to abundant charged species. However, the maximum box size allowed for AIMD by currently available computation power can hardly contain a full EDL.^218^ Computational efficient machine learning (ML) force fields provides a promising way to solve this problem, although current ML force field framework should be adjusted to accommodate the applied EF or GC approach, where pioneer progress have been made already.^219,220^

Catalytic control via electrode-immobilization of molecular catalysts is a lofty goal that necessitates intense and widespread research. Judicious choice of each part of a system’s design (electrode, adsorbate, solvent, electrolyte) is necessary for optimization toward a specific process. Gaining a deep understanding of each of these effects warrants further fundamental research and requires joint efforts through chemical catalysis, *in situ* spectroscopic characterization, and theoretical-computational modeling.
